# Diphenylcarbene Protected by Four *ortho*-Iodine Groups: An Unusually Persistent Triplet Carbene

**DOI:** 10.3390/molecules21111545

**Published:** 2016-11-15

**Authors:** Katsuyuki Hirai, Kana Bessho, Kosaku Tsujita, Toshikazu Kitagawa

**Affiliations:** 1 Community-University Research Cooperation Center, Mie University, Tsu, Mie 514-8507, Japan; 2 Department of Chemistry for Materials, Graduate School of Engineering, Mie University, Tsu, Mie 514-8507, Japan; kinou@chem.mie-u.ac.jp (K.B.); cultivation_eigode@i.softbank.jp (K.T.)

**Keywords:** triplet carbene, diazo compound, photolysis, steric hindrance, substituent effect

## Abstract

Diphenyldiazomethane with four iodine groups at the *ortho* positions and two *tert*-butyl groups at the *para* positions, i.e., bis(4-*tert*-butyl-2,6-diiodophenyl)diazomethane (**1a**-N_2_), was synthesized as a sterically hindered triplet carbene precursor. Irradiation of **1a**-N_2_ in solution effectively generated the corresponding triplet diphenylcarbene ^3^**1a**, which was characterized by UV-vis spectroscopy at low temperature, along with laser flash photolysis techniques at room temperature. The UV-vis spectrum of ^3^**1a** was obtained by irradiating **1a**-N_2_ in a 2-methyltetrahydrofuran matrix at 77 K. The ESR spectrum showed no triplet carbene signals, while a radical species was observed at the anticipated temperature of the decomposition of triplet carbene ^3^**1a**. Transient absorption bands ascribable to ^3^**1a** were observed by laser flash photolysis of **1a**-N_2_ in a degassed benzene solution and decayed very slowly with a second-order rate constant (2*k/*ε*l*) of 5.5 × 10**^−^**^3^·s**^−^**^1^. Steady-state irradiation of **1a**-N_2_ in degassed benzene afforded 9,10-diarylphenanthrene derivative **2a** in a 31% yield. Triplet carbene ^3^**1a** was also trapped by either oxygen (*k*_O2_ = 6.5 × 10^5^ M**^−^**^1^·s**^−^**^1^) or 1,4-cyclohexadiene (*k*_CHD_ = 1.5 M**^−^**^1^·s**^−^**^1^) to afford the corresponding ketone **1a**-O or the diarylmethane **1a**-H_2_. The carbene was shown to be much less reactive than the triplet diphenylcarbene that is protected by two *ortho*-iodo and two *ortho*-bromo groups, ^3^**1b**.

## 1. Introduction

Although stable *N*-heterocyclic carbenes (NHCs) have been frequently used as effective ligands for transition-metal catalysts [1,2], the instability of their triplet counterparts has hampered their use as materials. Thus, the stabilization and isolation of a triplet carbene has emerged as the next challenging target. Recent growing interest in triplet carbenes as potential organic ferromagnets [3,4,5] has added a more practical aspect to the project. Steric protection is an ideal method to achieve sufficient stabilization of a triplet carbene with its electronic integrity (one centered diradical) intact. Based on this concept, we generated many diphenylcarbenes that have various substituents at the ortho positions [6,7,8,9]. The strategy, however, encounters limitations when alkyl or aryl groups are employed as protecting groups. The central carbon atom of carbenes abstracts hydrogen even from very poor sources of electrons such as C-H bonds. Thus, the *tert-*butyl group, which has been successfully used to protect many reactive centers, is almost powerless to stabilize a triplet carbene center [10].

On the other hand, the van der Waals radius of bromine atom (185 pm) is larger than that of methyl group (175 pm) [11], and furthermore, the C-Br bond length (181 pm) of bromobenzene is longer than that of the C-CH_3_ (177 pm) in toluene [11]. Thus, the bromine groups at *ortho* positions on diphenylcarbene overhang the carbene carbon more effectively. Moreover, halides involving bromine atom are not reactive with the triplet state [12,13,14]. For example, we previously showed that triplet bis(2,6-dibromo-4-*tert*-butylphenyl)carbene (^3^**1c**) had a half-life of 16 s in a benzene solution at room temperature [15,16].

**Scheme 1:**
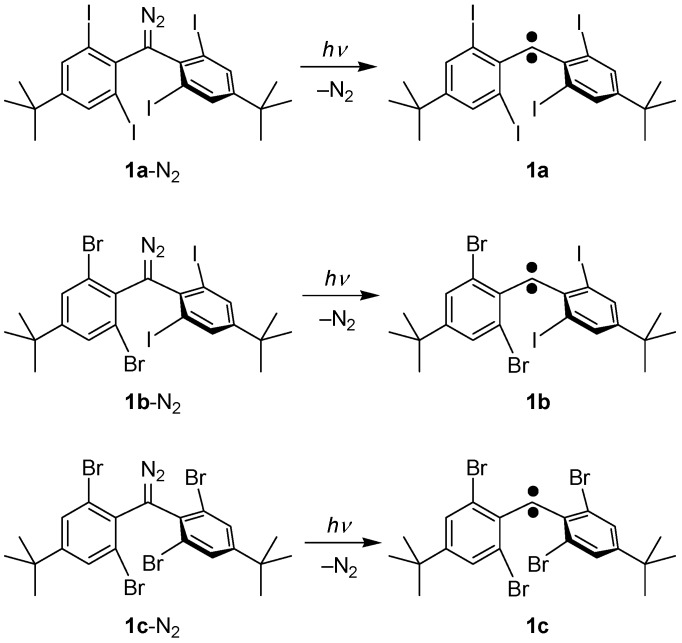
Generation of diarylcarbenes **1**.

The iodine atom has been regarded as a better kinetic protector of carbene because its van der Waals radius (198 pm) is much larger than that of the bromine atom (185 pm) [11]. In addition, according to theoretical calculations [17,18], the length of the C-I bond (210 pm) of iodobenzene is greater than that of the C-Br bond (189 pm) in bromobenzene. Recently, we generated and characterized a triplet diphenylcarbene bearing two iodine and two bromine groups at the *ortho* positions, (2,6-dibromo-4-*tert*-butylphenyl)(4-*tert*-butyl-2,6-diiodophenyl)carbene ^3^**1b** [19] (Scheme 1). This species was found to be a persistent triplet carbene, but contrary to our expectations, its half-life (*t*_1/2_ = 24 s) was only 1.5 times longer than that of ^3^**1c**. The bimolecular rate constants of the decay of ^3^**1b** by triplet carbene quenchers were also only moderately smaller than those of ^3^**1c**. Although an iodine group shielded the carbene center more effectively than a bromine group, the half-occupation of an iodine atom at the four *ortho* positions of a diphenylcarbene was insufficient to protect the carbene center. One may expect that an extreme stabilization would be achieved by thorough substitution of the four *ortho* positions with iodo groups. In the present study, we were successful in preparing a precursor diphenyldiazomethane with four *ortho*-iodo groups, bis(4-*tert*-butyl-2,6-diiodophenyl)diazomethane (**1a**-N_2_). Here we report that triplet diphenylcarbene ^3^**1a** generated by the photolysis of **1a**-N_2_, is an unusually persistent triplet diphenylcarbene with an extended half-life of 18.4 min.

## 2. Results and Discussion

### 2.1. Preparation of Precursor Diazomethane

**Scheme 2:**
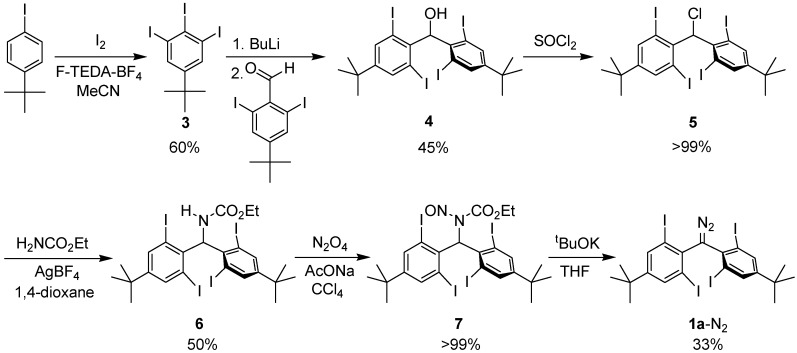
Synthesis of bis(2,6-diiodo-4-*tert*-butylphenyl)diazomethane (**1a**-N_2_).

Most diphenyldiazomethanes that bear four kinetic protectors at the *ortho* positions have been prepared from the corresponding diphenylmethanols via the carbamate derivatives [10,15,16,19]. For the synthesis of **1a**-N_2_ (Scheme 2), the starting alcohol was expected to be obtained by a coupling reaction of a 2,6-diiodobenzaldehyde with a 2,6-diiodophenyllithium. According to the synthesis plan, we attempted the lithiation of 5-*tert*-butyl-1,2,3-triiodobenzene (**3**), of which the *tert*-butyl group affords a *para*-substituent in **1a**. A *para*-*tert*-butyl group is known to serve as a good protecting group in diphenylcarbenes [15,16]. Fortunately, the lithiation proceeded exclusively at the 2-position of triiodobenzene to selectively afford the corresponding diphenylmethanol **4** by the subsequent reaction with 4-*tert*-butyl-2,6-diiodobenzaldehyde [19]. The success of the synthesis of alcohol **4** opened the way to diazomethane **1a**-N_2_. Alcohol **4** was chlorinated, substituted with ethyl carbamate, nitrosated, and treated with potassium *tert*-butoxide to give the crude diazomethane. The purification by preparative thin layer chromatography (alumina, hexane as eluent) afforded pure **1a**-N_2_ as yellow crystals in a 33% yield. Diazo compound **1a**-N_2_ was very stable for a diazomethane and did not decompose in a refrigerator (at −20 °C) over an extended period of time.

### 2.2. Spectroscopic Studies

#### 2.2.1. ESR Studies in Rigid Matrix at Low Temperature

**Figure 1:**
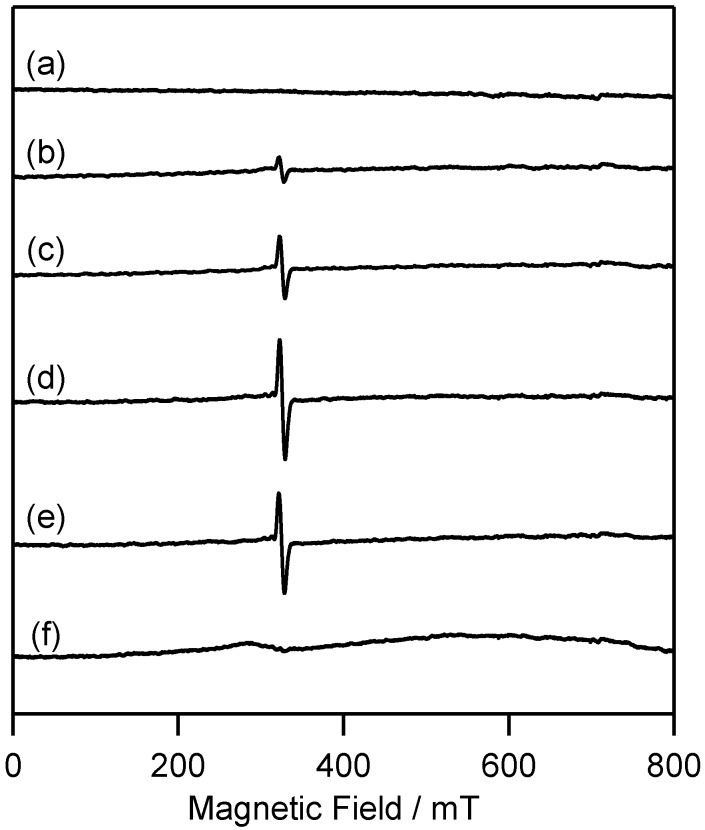
(**a**) ESR spectrum obtained by irradiation (λ > 350 nm) of diazo compound **1a**-N_2_ in 2-methyltetrahydrofuran at 77 K; (**b**–**e**) The same sample measured at 77 K after thawing to 200 K (**b**), 220 K (**c**), 230 K (**d**), and 300 K (**e**); (**f**) The same sample measured at 77 K after exposure to air.

Most sterically hindered diphenylcarbenes are known to give rise to stable triplet ESR signals by irradiation of the corresponding diphenyldiazomethanes in 2-methyltetrahydrofuran (2-MTHF) at 77 K [10,15,16,20,21,22,23,24]. On the other hand, the signals of triplet carbene ^3^**1b** with two iodine atoms were invisible under the same conditions [19]. Similar invisibilities were observed in the ESR measurements of 2-iodophenylnitrene and (2-iodophenyl)(phenyl)carbene, and were explained by extreme broadening of the signal due to orbital degeneracy and strong spin-orbit coupling in the iodine atoms [25]. As expected from these facts, photolysis (λ > 350 nm) of diazomethane **1a**-N_2_ in a low temperature 2-MTHF matrix afforded no triplet signals due to carbene **1a** (Figure 1a). The influence of the iodine atoms on ESR line broadening may be larger than that in **1b** owing to the presence of four iodo groups.

**Scheme 3:**
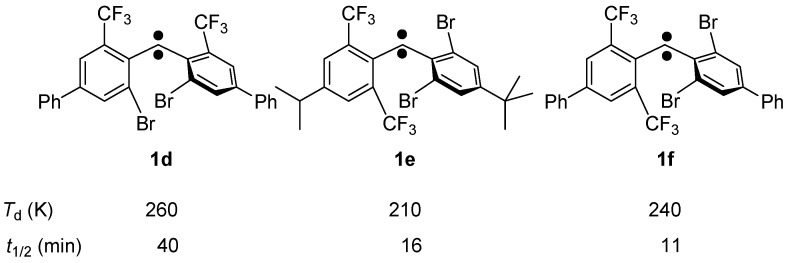
Diphenylcarbenes **1d**–**f** protected by two *ortho*-trifluoromethyl and two *ortho*-bromo groups.

We previously reported that the ESR signals of carbene **1c** with four *ortho*-bromo groups were observed in a 2-MTHF matrix at 77 K and disappeared irreversibly by warming the matrix to 170 K [26]. On the other hand, triplet carbene ^3^**1b** was invisible under the same conditions, and a new doublet signal at 327 mT due to carbon radical species appeared by warming up to 160 K, which implies the decomposition of ^3^**1b** [19]. This means that the triplet carbene ^3^**1b** abstracts a hydrogen from 2-MTHF of the matrix to form the corresponding diphenylmethyl radical. If carbene **1a** decays by a similar form of degradation, a doublet signal of the corresponding radical species would appear when the matrix is warmed up. To confirm this assumption, ESR measurements were carried out after warming the matrix involving the photoproducts and then recooling to 77 K to compensate for the weakening of signals due to the Curie law [27,28,29]. Although gradual warming of the photolyzed sample up to 190 K gave no ESR signals, a new doublet signal appeared at 327 mT at 200 K (Figure 1b). The signal intensity increased gradually as the sample was warmed up to 230 K, and decreased only slightly at 300 K (Figure 1c–e). The signal disappeared when the 2-MTHF solution was exposed to air (Figure 1f). The appearance of the doublet signal can be attributed to the formation of bis(4-*tert*-butyl-2,6-diiodophenyl)methyl radical via hydrogen abstraction. We used the temperature (*T*_d_) at which signals due to a triplet carbene disappear as a measure of the thermal stability of triplet carbenes [9]. If ^3^**1a** is assumed to abstract a hydrogen from the solvent molecule at *T*_d_, ^3^**1a** (*T*_d_ = 200 K) is considered much more resistant to degradation than **1b** and **1c** (*T*_d_ = 170 and 160 K, respectively). We previously reported that diphenylcarbenes protected by two bromine and two trifluoromethyl groups, **1d**–**f**, are known to be the unusually persistent diphenylcarbenes with *T*_d_s of 210–260 K (Scheme 3) [20,21]. The present result indicates that carbene **1a** is also an unusually persistent diphenylcarbene with a stability comparable to **1e**.

#### 2.2.2. Magnetic-Susceptibility Measurements

To obtain evidence of the ground triplet spin state of carbene **1a**, the magnetic susceptibility of the photoproduct of diazomethane **1a**-N_2_ was measured. A 2-MTHF solution of **1a**-N_2_ (2.0 mM) was placed inside the sample compartment of a superconducting quantum interference device (SQUID) magnet/susceptometer and was irradiated at 10 K with a laser light at 442 nm. The development upon magnetization was measured at 5 K in a constant field of 5000 Oe. The magnetization values gradually increased with the irradiation time and reached a plateau after 12 h. After a plateau was established, the magnetization values, *M*_a_, were measured at 5.0 K in a range of 0–50,000 Oe. The net magnetization generated by the irradiation of **1a**-N_2_ was estimated from the difference between *M*_a_ and the corresponding magnetization values, *M*_b_, obtained before irradiation. The plot of the magnetization (*M*) versus the magnetic field (*H*) was analyzed in terms of the Brillouin function as follows [30,31,32,33,34,35]:

*M* = *M*_a_ − *M*_b_ = *FNgJμ*_B_*B*(*χ*) (1)

where *F* is the generation factor for carbene estimated from saturated magnetization, *N* is the number of the molecule, *g* is the Landé *g*-factor, *J* is the quantum number for the total angular momentum, and *μ*_B_ is the Bohr magneton. Since diphenylcarbene is composed of light elements, the orbital angular momentum should be negligible, and *J* can be replaced with the spin quantum number *S*. The *M* vs. *H* plot is shown in Figure S1. The observed data was best fitted to Equation (1) with *S* = 0.93 (*F* = 0.35) at 5.0 K. Although this *S* value is somewhat smaller than the theoretical value (*S* = 1) for a triplet carbene, this result clearly demonstrates the generation of a triplet species, i.e., carbene **1a**, by the photolysis of **1a**-N_2_.

#### 2.2.3. UV-Vis Studies in Rigid Matrix at Low Temperature

**Figure 2:**
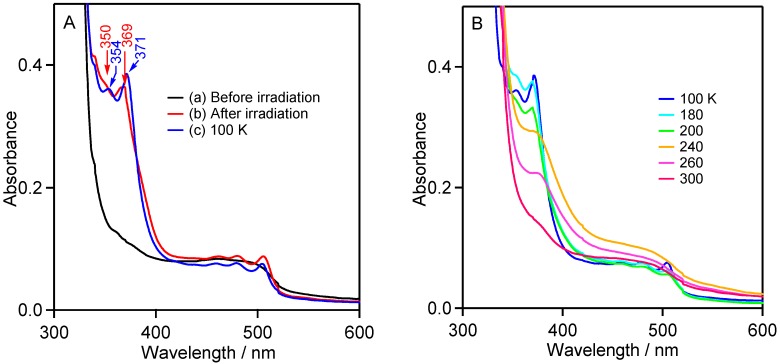
UV-vis spectra obtained by irradiation (λ > 350 nm) of diazo compound **1a**-N_2_ in 2-methyltetrahydrofuran at 77 K: (**A**) (a) Spectrum of **1a**-N_2_, (b) The same sample after irradiation, (c) The same sample after thawing to 100 K; (**B**) UV-vis spectral change measured upon thawing the same sample from 100 to 300 K.

Photolysis (λ > 350 nm) of **1a**-N_2_ in a 2-MTHF matrix at 77 K afforded new absorption bands, strong maxima at 350 and 369 nm and broad and weak bands at 462, 480 and 506 nm, at the expense of the precursor (Figure 2A(a,b)). Most of the sterically hindered triplet diphenylcarbenes in 2-MTHF matrix are known to show strong absorption bands in the UV region (320–360 nm) and broad and weak bands in the visible region (430–510 nm) [9]. The absorption bands disappeared irreversibly when the matrix was warmed up to room temperature. We assigned the species having these absorption bands to triplet carbene ^3^**1a** on the basis of similarity in the spectrum and in thermal behavior of ^3^**1b** [19].

The thermal stability of triplet carbene ^3^**1a** was then estimated by warming the matrix. When warmed to 100 K, the absorption maxima of the bands due to ^3^**1a** shifted from 350 and 369 nm to 354 and 371 nm, respectively (Figure 2A(c)). The spectral changes can be attributed to a decrease in the viscosity of the 2-MTHF matrix, because similar changes in other sterically hindered diarylcarbenes are interpreted in terms of geometrical changes in the molecules due to a sudden decrease in the viscosity of 2-MTHF from 10^7^ to 10^3^ P [36]. The bands of ^3^**1a** started to decrease slowly at 180 K and disappeared irreversibly at 240 K (Figure 2B). The temperature at which the bands of ^3^**1a** started to decrease was roughly in accordance with the emergence of the ESR signal of the radical.

Diphenylcarbenes with no substituents at the ortho positions in a 2-MTHF matrix disappear between 100 and 105 K [7]. Carbene ^3^**1b** is protected by two bromo and two iodo groups, but it started to decay at 150 K in 2-MTHF [19]. The even higher decay temperature of **1a** clearly demonstrates that it is much more stable than ^3^**1b**.

#### 2.2.4. Laser Flash Photolysis Studies in Solution at Room Temperature

For quantitative evaluation of the stability of carbene **1a**, the lifetime measurement was carried out in degassed benzene at 25 °C, which we ordinarily use for the lifetime measurement of sterically congested diarylcarbenes [6,7,8,9]. The lifetime of **1a** was too long to monitor by the laser flash photolysis (LFP) apparatus that we routinely use. Thus, the lifetime was estimated using a conventional UV-vis spectroscopic method [20,21,37,38].

**Figure 3:**
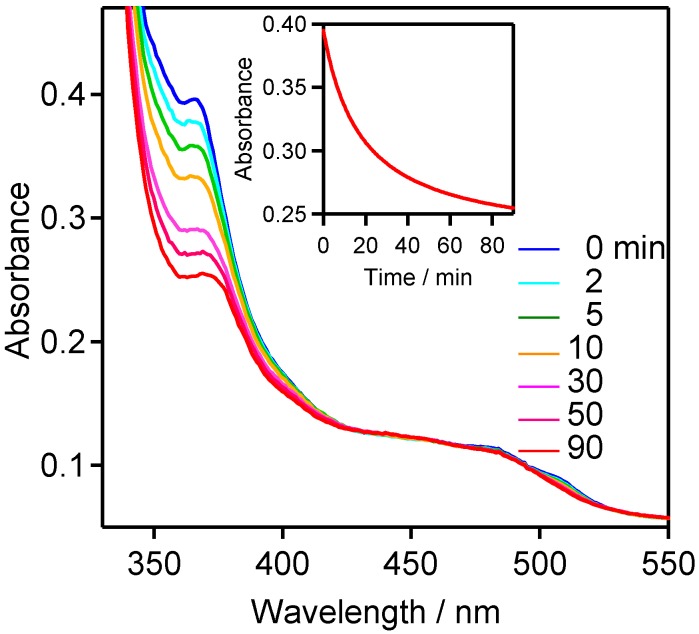
Absorption of transient products formed during laser irradiation (λ = 308 nm) of **1a**-N_2_ in degassed benzene at 25 °C recorded from immediately after irradiation to 90 min after irradiation. The inset shows the time course of the absorption monitored at 366 nm.

Laser pulse photolysis at 308 nm of **1a**-N_2_ in degassed benzene at 25 °C produced a transient species showing maxima at 366, 480, and 506 nm, which appeared coincidentally with the laser pulse (Figure 3). These absorption bands were essentially similar to those observed in the photolysis of **1a**-N_2_ in 2-MTHF at 77 K, although the maxima were slightly shifted. The transient bands were assigned to triplet ^3^**1a**, since the bands were quickly quenched by oxygen to give the corresponding diphenyl ketone oxide (*vide infra*). The absorption bands decayed very slowly but did not disappear completely even after 1.5 h. The decay was found to be a second-order process (2*k*/ε*l* = 5.5 × 10**^−^**^3^·s**^−^**^1^) with a half-life of 18.4 min (Figure 3 inset, Table 1). The kinetic data indicates that ^3^**1a** is extremely persistent compared with that of ^3^**1b** (2*k*/ε*l* = 0.17 s**^−^**^1^, *t*_1/2_ = 24 s) [19] and ^3^**1c** (2*k*/ε*l* = 0.35 s**^−^**^1^, *t*_1/2_ = 16 s) [15,16].

Since triplet carbenes are easily trapped with oxygen or 1,4-cyclohexadiene (CHD) [39], these trapping reactions can be employed as a quantitative scale of the reactivities of the triplet carbenes.

**Scheme 4:**
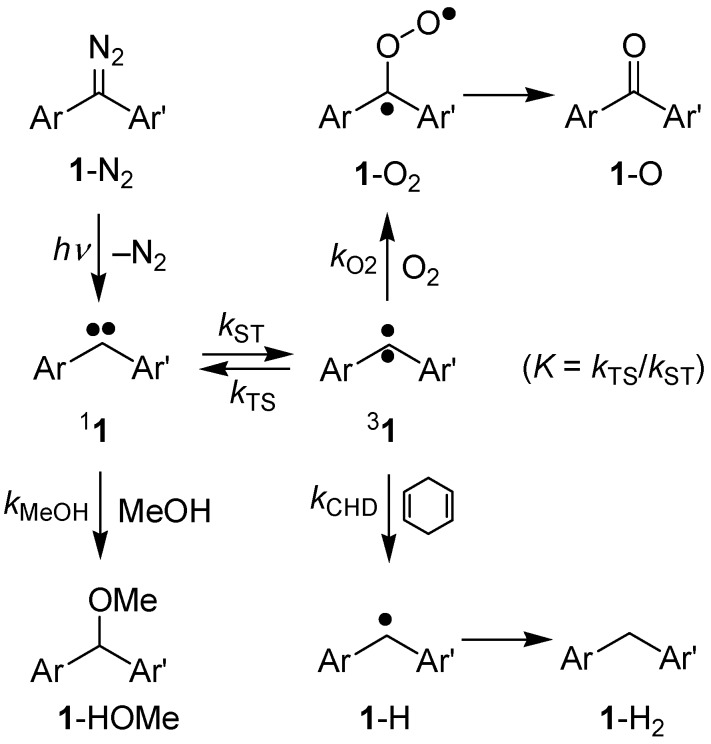
Photolysis of diazo compounds **1**-N_2_ in the presence of carbene quenchers.

The LFP (λ = 308 nm) of **1a**-N_2_ in O_2_ saturated benzene at 25 °C resulted in a rapid decay of carbene ^3^**1a** and a concurrent appearance of a broad absorption band at around 415 nm (Figure S2A(b)), which was clearly different from the band of **1a** (Figure S2A(a)). In addition, the solution after the LFP was found to contain the corresponding benzophenone **1a**-O as a major product. It is well documented that the oxygen trapping of diphenylcarbenes generates the corresponding benzophenone oxides with a broad absorption band at around 390–450 nm to afford the ketone derivative (Scheme 4) [40,41,42,43,44,45,46,47]. Thus, our observations suggested that carbene ^3^**1a** was rapidly trapped with oxygen to form the ketone oxide **1a**-O_2_ like most of diphenylcarbenes. The rate constant (*k*_O2_) for the trapping of ^3^**1a** with oxygen was estimated to be 6.5 × 10^5^ M^−1^·s^−1^ based on a plot of the observed growth rate of **1a**-O_2_ as a function of the oxygen concentration (Figure S2B, Table 1). The rate constant is one order of magnitude smaller than that observed with ^3^**1b** (*k*_O2_ = 7.3 × 10^6^ M^−1^·s^−1^) [19] and 30 times smaller than that of ^3^**1c** (*k*_O2_ = 2.1 × 10^7^ M^−1^·s^−1^) [16].

The LFP (λ = 308 nm) of **1a**-N_2_ in degassed benzene containing 1,4-cyclohexadiene gave a new species with an absorption band at 380 nm. The diphenylmethyl radicals, formed as a result of a hydrogen abstraction of the carbenes **1b**,**c** from 1,4-cyclohexadiene, showed absorption maxima at wavelengths 20–30 nm longer than that of the precursor carbene bands (ca. 350 nm) [16,19]. Thus, this species, generated from **1a**-N_2_, was also assigned to the corresponding diphenylmethyl radical (**1a**-H) (Scheme 4). The spent solution contained the diphenylmethane **1a**-H_2_ as the main product. Owing to the overlap between the absorption band of the radical and the tail of the carbene, the growth curves of the radical were not observed clearly (Figure S3A). However, a linear plot was obtained between the observed pseudo-first-order rate constants of the decay of the carbene monitored at 350 nm and [CHD] (Figure S3B), and its slope gave the rate constant (*k*_CHD_) for the reaction of ^3^**1a** with the diene, 1.5 M^−1^·s^−1^, which is 1 and 2 orders of magnitude smaller than those observed with triplet diiododibromo ^3^**1b** (39 M^−1^·s^−1^) [19] and tetrabromo ^3^**1c** (5.3 × 10^2^ M^−1^·s^−1^) [15,16], respectively (Table 1).

The LFP of **1a**-N_2_ in a degassed benzene–methanol mixed solvent also formed triplet carbene ^3^**1a**. The observed decay rate constant (*k*_obs_) of ^3^**1a** increased as the fraction of methanol increased. However, the plot of *k*_obs_ vs methanol concentration was not linear in the concentration range of 2.47 to 9.88 M. The trapping of some sterically hindered diarylcarbenes with methanol have been reported to show better linearities when the plot was performed against the square of the methanol concentration [19,48]. Carbene **1a** also showed a more linear plot when *k*_obs_ was plotted against [MeOH]^2^, and the trapping rate constant (*Kk*_MeOH_) was 0.033 M^−2^·s^−1^ as estimated from the slope of this plot (Figure S4, Table 1). This is one order of magnitude smaller than the rate constants observed with ^3^**1b** and ^3^**1c** (0.30 and 0.42 M^−2^·s^−1^, respectively [19]).

The insertion of carbenes to the O-H bond of alcohols to form ethers is usually considered to occur from the singlet states of these species (Scheme 4). The decrease in *Kk*_MeOH_ may be caused either by an increased energy gap between the ground triplet and singlet states or by the deceleration of the O-H insertion. Since theoretical studies have suggested that the energy gap for **1a** is only slightly larger than those for **1b** and **1c** (*vide infra*), the difference in the rate constants (*Kk*_MeOH_) is mainly controlled by the rate (*k*_MeOH_) of the insertion reaction of singlet carbenes to methanol, rather than by the singlet-triplet equilibrium constant (*K*). Thus, the *ortho*-iodine groups have a significant rate-retarding effect on the insertion reaction of the singlet carbenes. Thus, carbene ^3^**1a** showed a higher stability estimated in degassed benzene and in degassed 2-MTHF and lower reactivities toward triplet carbene quenchers (oxygen and 1,4-cyclohexadiene) and a singlet carbene quencher (methanol) than the bromine-substituted carbenes ^3^**1b** and ^3^**1c**.

### 2.3. Product Analysis Studies

Although steric protection is an efficient method for stabilization of the triplet diphenylcarbenes, complete protection of triplet carbenes has not been achieved, because even such carbenes decay mainly by dimerization. When long-lived diphenylcarbenes are generated in degassed benzene in the absence of carbene quenchers at room temperature, most are known to dimerize at the carbenic center to form the corresponding tetraphenylethenes as the main product. For example, dimesitylcarbene and bis(2,4,6-trichlorophenyl)carbene produced tetramesitylethene [10] and tetrakis(2,4,6-trichlorophenyl)ethene [22] in high yields, respectively. On the other hand, the dimerization product from bis(2,6-dibromophenyl)carbenes underwent photocyclization to give the corresponding phenanthrene derivatives [15,16]. By the same token, carbene **2b** afforded the corresponding phenanthrene derivative with the concomitant elimination of an iodine molecule [19].

**Scheme 5:**
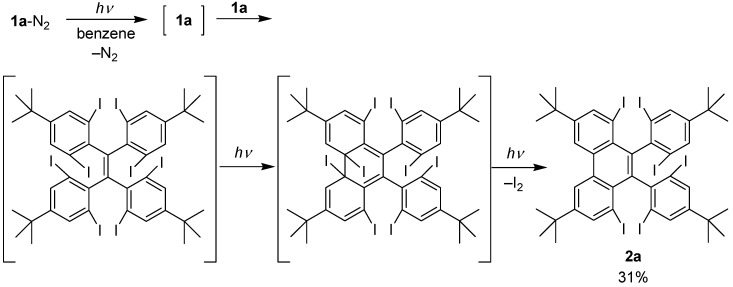
Photolysis of diazo compound **1a**-N_2_ in degassed benzene-*d*_6_ at 25 °C.

Photolysis (λ > 350 nm) of **1a**-N_2_ (5.0 mg) in degassed benzene-*d*_6_ (0.5 mL) in a sealed NMR tube at 25 °C was monitored by ^1^H-NMR spectroscopy. After 50 min irradiation, very weak ^1^H-NMR signals at 9.03, 8.82, 7.84, 1.23, and 1.09 ppm, along with weak and broad peaks were observed at the expense of the signals of **1a**-N_2_. The ^1^H-NMR spectrum of a fraction separated by a recycled gel permeation chromatography (GPC) of the resultant mixture showed the phenanthrene derivative **2a** (a brown oil in a 31% yield) (Scheme 5). During the photolysis, it is reasonable that **2a** was formed as a result of dimerization at the carbene center followed by photocyclization and deiodination. The ^1^H-NMR spectra of other GPC fractions showed no detectable peaks. The yield of **2a** was higher than that of **2b** (18%) but lower than that of **2c** (48%). The carbene dimer intermediate may have difficulty in forming from **1a** compared to from **1c** due to the steric repulsion between the bulky iodo groups.

**Scheme 6:**
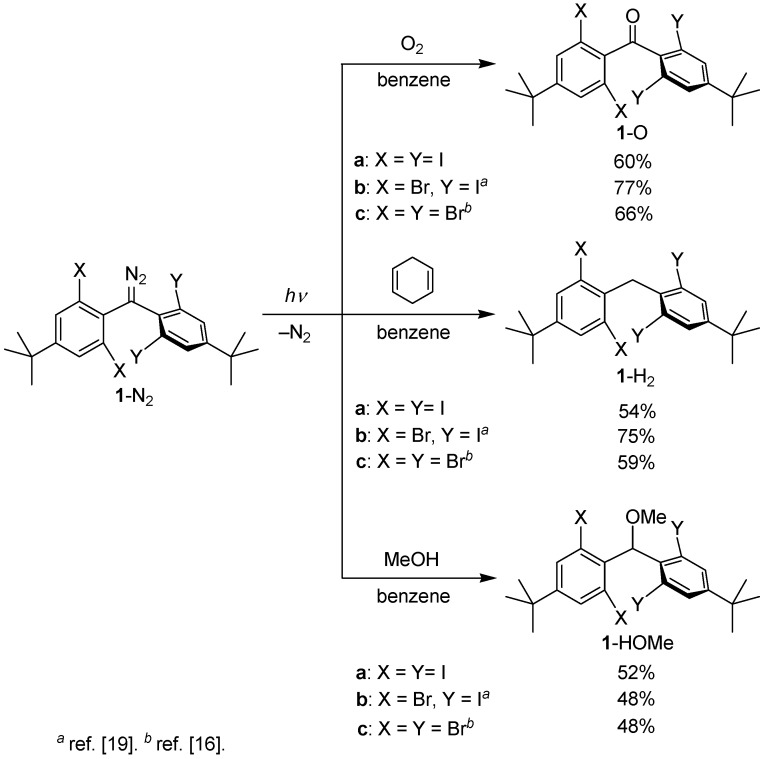
Photoproducts obtained by irradiation of diazo compound **1**-N_2_ at 25 °C.

Photolysis (λ > 350 nm) of **1a**-N_2_ in an O_2_-saturated benzene solution afforded tetraiodobenzophenone **1a**-O as the sole product in a 60% yield. On the other hand, irradiation (λ > 350 nm) of **1a**-N_2_ in degassed benzene in the presence of 1,4-cyclohexadiene gave diphenylmethane **1a**-H_2_ in a 54% yield. Similar irradiation in degassed benzene–methanol afforded methyl ether **1a**-HOMe (52%) (Scheme 6). We previously showed that the irradiation of **1b**-N_2_ and **1c**-N_2_ in the presence of the carbene quenchers afforded ketones **1**-O, diphenylmethanes **1**-H_2_, and methyl ethers **1**-HOMe in good yields [16,19]. Thus, the lifetimes of carbenes **1** hardly affected the product yields.

### 2.4. DFT Calculations

**Figure 4:**
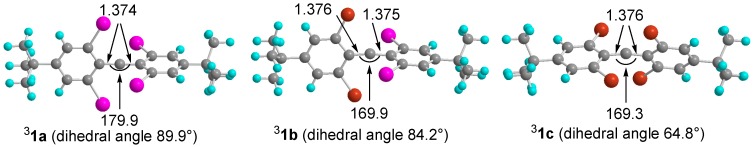
Calculated (M05/3–21G for I atom and 6–31G* for other atoms) structures and selected bond distances (in Å) and angles (in degrees) of triplet carbenes ^3^**1a**, ^3^**1b**, and ^3^**1c**.

To obtain direct insight into the differences among the structures of carbenes **1a**, **1b**, and **1c**, their singlet and triplet states were optimized by DFT calculations [18]. The optimized geometries at the M05/6–31G* level of theory (3–21G level for iodine atom) indicated that the carbene-center angle/dihedral angle between two phenyl rings of singlet ^1^**1a** are 130.2°/80.1°, but those for triplet ^3^**1a** are 179.9°/89.9° (Figure 4), indicating that triplet ^3^**1a** has a linear structure with two phenyl rings more orthogonal to each other compared to those in ^1^**1a**. On the other hand, the dihedral angles in triplet ^3^**1b** and ^3^**1c** were significantly smaller than that in **1a**, enabling the optimal shielding of the carbene center in **1a**. The C-C distances between the aromatic and carbene carbons are slightly shorter for ^3^**1a** (1.374 Å) than for either ^3^**1b** (1.375 and 1.376 Å) or ^3^**1c** (1.376 Å). The spin density on the carbene carbon (1.48 for ^3^**1a**) are similar to those of ^3^**1b** and ^3^**1c** (1.48 and 1.51). For **1a**, the triplet state lies 16.6 kcal/mol below the singlet state. The energy gap between the singlet and triplet states is slightly larger than that for either carbene **1b** (Δ*E*_ST_ = 15.4 kcal/mol) or **1c** (Δ*E*_ST_ = 14.2 kcal/mol).

## 3. Materials and Methods

### 3.1. General

^1^H- (300 MHz) and ^13^C-NMR (75.5 MHz) spectra were determined with a JNM-AL300 FT/NMR spectrometer (JEOL, Tokyo, Japan) in CDCl_3_ with Me_4_Si as an internal reference. ^13^C-NMR (100 MHz) spectra were obtained using a JNM-ECX400 FT/NMR spectrometer (JEOL, Tokyo, Japan). The NMR spectra were shown in Supplementary materials. IR spectra were measured either with films on a NaCl plate or with KBr pellets using a FT/IR-410 spectrometer (JASCO, Tokyo, Japan). UV-vis spectra were measured with a JASCO V-560 or MaltiSpec-1500 spectrometer (Shimadzu, Kyoto, Japan). The mass spectra were recorded on a JEOL JMS-600H mass spectrometer or a Voyager DE-Pro MALDI-TOF mass spectrometer (Applied Biosystems, Foster City, CA, USA). Thin layer chromatography for diazo compounds was performed using aluminum oxide 60 PF_254_ on a glass plate (Type E) (Merck, Tokyo, Japan). Column chromatography was performed using silica gel (63–210 μm) or neutral alumina (Act. I, inactivated with 5% water) for diazo compound. Recycle GPC was undertaken with a JASCO PU-2086 chromatograph with a UV-2070 UV-vis detector using a GPC H-2001 (20 mm × 50 cm) column (Shodex, Tokyo, Japan).

### 3.2. Synthesis of Bis(4-tert-butyl-2,6-diiodophenyl)diazomethane (***1a**-N_2_*)

*5-tert-Butyl-1,2,3-triiodobenzene* (**3**): To a solution of 4-*tert*-butyl-1-iodobenzene (2.50 g, 9.61 mmol) in dry MeCN (100 mL) was added I_2_ (8.50 g, 33.5 mmol) and 90% F-TEDA-BF_4_ (6.72 g, 33.5 mmol), and the reaction mixture was stirred at 65 °C for 14 h. The solvent was removed under reduced pressure and the crude mixture was dissolved in CH_2_Cl_2_ (90 mL). After the insoluble material was filtered off, the solution was washed with saturated aqueous Na_2_S_2_O_3_·5H_2_O and brine, and dried over anhydrous Na_2_SO_4_. The solvent was evaporated, and the starting iodobenzene and by-product, 4-*tert*-butyl-1,2-diiodobenzene were removed using a glass tube oven (160 °C/3.0 Torr). The residue was chromatographed through a column of silica gel using hexane as an eluent to give 5-*tert*-butyl-1,2,3-triiodobenzene (**3**) as yellow crystals (1.03 g, 60%): ^1^H-NMR (CDCl_3_, ppm) δ 7.82 (s, 2H), 1.25 (s, 9H) [49].

*Bis(4-tert-butyl-2,6-diiodophenyl)methanol* (**4**): To a solution of 5-*tert*-butyl-1,2,3-triiodobenzene (**3**) (1.01 g, 2.0 mmol) in dry ether (40 mL) was added BuLi (2.64 M in hexane, 0.80 mL, 2.1 mmol) at –78 °C. The solution was stirred for 6 h, and then a solution of 4-*tert*-butyl-2,6-diiodobenzaldehyde (0.86 g, 2.1 mmol) in dry ether (7 mL) was added dropwise over 3 min at −78 °C. The mixture was stirred at room temperature for 20 h and was then quenched by saturated aqueous ammonium chloride (25 mL). The mixture was extracted with ether, and the organic layer was washed with water and dried over anhydrous Na_2_SO_4_. The solvent was removed under reduced pressure, and the residue was chromatographed through a column of silica gel using hexane–chloroform (2:1) as an eluent to give bis(4-*tert*-butyl-2,6-diiodophenyl)methanol (**4**) as colorless crystals (0.71 g, 45%): m.p. 77.7–80.6 °C; ^1^H-NMR (CDCl_3_, ppm) δ 7.94 (s, 4H), 6.19 (d, *J* = 8.8 Hz, 1H), 2.76 (d, *J* = 8.8 Hz, 1H), 1.28 (s, 18H); ^13^C-NMR (CDCl_3_, ppm) δ 154.0, 139.6, 137.7, 99.5, 86.9, 34.1, 31.0; HRMS calcd. for C_21_H_23_I_4_O [M – H]^+^ 798.7923, found *m*/*z* 798.7922.

*Ethyl N-[bis(4-tert-butyl-2,6-diiodophenyl)methyl]carbamate* (**6**): A solution of alcohol **4** (472 mg, 590 μmol) and thionyl chloride (9 mL, 0.12 mol) was stirred at room temperature for 6 h, and the mixture was evaporated under reduced pressure to give bis(4-*tert*-butyl-2,6-diiodophenyl)chloromethane (**5**) as orange crystals (480 mg). This compound was used in the next experiment without further purification: ^1^H-NMR (CDCl_3_, ppm) δ 7.98 (s, 4H), 6.64 (s, 1H), 1.28 (s, 18H). To a mixture of silver tetrafluoroborate (110 mg, 567 μmol) and ethyl carbamate (2.50 g, 28.1 mmol), heated at 60 °C, was added a solution of chloromethane **5** (250 mg, 313 μmol) in 1,4-dioxane (3 mL), and the resultant mixture was heated at 100 °C for 20 h. After cooling at room temperature, the mixture was filtered, and CHCl_3_ (20 mL) was added to the filtrate. The organic layer was washed well with water, dried over anhydrous Na_2_SO_4_, and the solvent was removed under reduced pressure. The residue was chromatographed through a column of silica gel using hexane–dichloromethane (1:5) as an eluent to give ethyl *N*-[bis(4-*tert*-butyl-2,6-diiodophenyl)methyl]carbamate (**6**) as yellow crystals (143 mg, 50%): m.p. 105.2–107.1 °C; ^1^H-NMR (CDCl_3_, ppm) δ 7.92 (s, 4H), 6.23 (d, *J* = 8.5 Hz, 1H), 5.18 (d, *J* = 8.5 Hz, 1H), 4.24 (q, *J* = 7.2 Hz, 2H), 1.31–1.27 (m, 21H); ^13^C-NMR (CDCl_3_, ppm) δ 154.2, 153.8, 139.9, 136.0, 100.3, 71.5, 61.4, 34.0, 30.9, 15.0; HRMS calcd. for C_24_H_30_I_4_NO_2_ [M + H]^+^ 871.8450, found *m*/*z* 871.8410.

*Bis(4-tert-butyl-2,6-diiodophenyl)diazomethane* (**1a**-N_2_): Into a stirred solution of carbamate **6** (189 mg, 217 μmol) in anhydrous CCl_4_ (15 mL) at −10 °C was bubbled dinitrogen tetroxide gas (3.00 g, 31.0 mmol), and sodium acetate (1.27 g, 15.5 mmol) was added. The mixture was stirred at room temperature for 23 h after the addition. The mixture was poured into a cold saturated aqueous sodium carbonate solution and extracted with CCl_4_. The organic layer was washed with 5% aqueous Na_2_CO_3_ solution and brine, dried over anhydrous Na_2_SO_4_, and the solvent was removed under reduced pressure to give ethyl *N*-nitroso-*N*-[bis(4-*tert*-butyl-2,6-diiodophenyl)methyl]carbamate (**7**) as a yellow semisolid (195 mg); ^1^H-NMR (CDCl_3_, ppm) δ 7.88 (s, 4H), 6.57 (s, 1H) 4.63 (q, *J* = 7.0 Hz, 2H), 1.49 (t, *J* = 7.0 Hz, 3H), 1.26 (s, 18H). To a solution of nitroso compound **7** in anhydrous THF (13 mL), cooled at –15 °C, was added potassium *tert-*butoxide (183 mg, 1.63 mmol) at once under an Ar atmosphere. After stirring at room temperature for 42 h, the mixture was poured into water (50 mL) and extracted with ether (20 mL × 3). The organic layer was washed with water and brine, dried over anhydrous Na_2_SO_4_, and evaporated. The residue was purified by short column chromatography (neutral alumina, hexane) and preparative TLC (aluminum oxide, hexane) to give bis(4-*tert*-butyl-2,6-diiodophenyl)diazomethane (**1a**-N_2_) as yellow crystals (58 mg, 33%): m.p. 187.6–189.2 °C (dec.); ^1^H-NMR (CDCl_3_, ppm) δ 7.94 (s, 4H), 1.29 (s, 18H); ^13^C-NMR (CDCl_3_, ppm) δ 153.8, 139.1, 131.9, 100.8, 77.7, 34.3, 31.0; IR (KBr disk, cm^–1^) 2059 (ν_C=N2_).

### 3.3. Product Analysis

All benzene solutions of diazo compounds were photolyzed through an UV-35 glass filter (AGC TECHNO GLASS, Shizuoka, Japan) with a 500 W Xe lamp (Wacom, Saitama, Japan) at 25 °C. Photolysis (λ > 350 nm) of **1a**-N_2_ (5.0 mg, 6.2 mmol) in degassed benzene-*d*_6_ (0.5 mL) for 50 min gave 3,6-di-*tert*-butyl-9,10-bis(4-*tert*-butyl-2,6-diiodophenyl)-1,8-diiodo-phenanthrene (**2a**): a brown oil (1.3 mg, 31% after recycle GPC separation) ; ^1^H-NMR (CDCl_3_, ppm) δ 8.81 (d, *J* = 2.0 Hz, 2H), 8.53 (d, *J* = 2.0 Hz, 2H), 7.66 (s, 4H), 1.50 (s, 18H), 1.30 (s, 18H); ^13^C-NMR (CDCl_3_, ppm) δ 154.3, 150.1, 143.9, 140.0, 136.7, 132.8, 128.1, 120.7, 108.2, 99.8, 92.9, 34.8, 34.2, 31.4, 31.2; MALDI-TOF-mass calcd. for C_42_H_44_I_6_ (M^+^) 1309.7706, found *m*/*z* 1309.7829.

Photolysis (λ > 350 nm) of **1a**-N_2_ (5.0 mg, 6.2 mmol) in O_2_-saturated benzene (1.0 mL) for 20 min gave bis(4-*tert*-butyl-2,6-diiodophenyl) ketone (**1a**-O) as colorless crystals (3.0 mg, 60% after recycle GPC separation); m.p. 219.5–220.4 °C; ^1^H-NMR (CDCl_3_, ppm) δ 7.94 (s, 4H), 1.30 (s, 18H); ^13^C-NMR (CDCl_3_, ppm) δ 195.9, 157.0, 139.4, 138.9, 97.1, 34.6, 30.9; IR (KBr, cm^–1^) 1669 (ν_CO_); MALDI-TOF-mass calcd. for C_21_H_22_I_4_O (M^+^) 797.7844, found *m*/*z* 797.7792.

Photolysis (λ > 350 nm) of **1a**-N_2_ (5.0 mg, 6.2 mmol) in degassed benzene (0.9 mL) in the presence of 1,4-cyclohexadiene (0.1 mL) for 20 min gave bis(4-*tert*-butyl-2,6-diiodophenyl)methane (**1a**-H_2_) as colorless crystals (2.6 mg, 54% after recycle GPC separation); m.p. 190.6–191.4 °C; ^1^H-NMR (CDCl_3_, ppm) δ 7.84 (s, 4H), 4.70 (s, 2H), 1.27 (s, 18H); ^13^C-NMR (CDCl_3_, ppm) δ 153.2, 139.4, 138.4, 101.7, 60.5, 34.1, 31.1; MALDI-TOF-mass calcd for C_21_H_23_I_4_ [M − H]^+^ 782.7973, found *m*/*z* 782.7956.

Photolysis (λ > 350 nm) of **1a**-N_2_ (5.0 mg, 6.2 mmol) in degassed benzene (0.5 mL) in the presence of methanol (0.5 mL) for 20 min gave bis(4-*tert*-butyl-2,6-diiodophenyl)methyl methyl ether (**1a**-HOMe) as colorless crystals (2.6 mg, 52% after recycle GPC separation); m.p. 226.4–227.3 °C; ^1^H-NMR (300 MHz, CDCl_3_, ppm) δ 7.97 (s, 4H), 5.64 (s, 1H), 3.46 (s, 3H), 1.29 (s, 18H); ^13^C-NMR (CDCl_3_, ppm) δ 154.4, 139.8, 135.2 100.4, 95.4, 59.2, 34.2, 31.1; MALDI-TOF-mass calcd. for C_22_H_25_ I_4_O [M − H]^+^ 812.8079, found *m*/*z* 812.8140.

### 3.4. ESR Measurements

The diazo compound was dissolved in 2-MTHF (1.5 × 10^–2^ M) and the solution was degassed in a quartz sample tube by five freeze-degas-thaw cycles. The sample was cooled in an optical transmission ESR cavity at 77 K and irradiated with a Wacom 500 W Xe lamp using a filter (λ > 350 nm). ESR spectra were measured on a JEOL JES TE 200 spectrometer (X-band microwave unit). The temperature was controlled by a 9650 microprocessor-based digital temperature controller (control ability within ±0.2 K).

### 3.5. SQUID Measurements

Magnetic susceptibility data were obtained on a MPMS-2A SQUID magnetometer/susceptometer (Quantum Design, Tokyo, Japan). Irradiation with light from a He-Cd laser (442 nm, 2074-M A03, Melles Griot, Saitama, Japan) through a flexible optical fiber was performed inside the sample room of the SQUID apparatus at 5–11 K. One end of the optical fiber was located 40 mm above the sample cell (capsule), and the other end was attached to a coupler for the laser. The bottom portion of the capsule (6 mm × 10 mm) was used as a sample cell. A quantity of 50 μL of the sample solution (1.0 mM) in 2-MTHF was placed in the cell that was held by a plastic straw. The irradiation was carried out until there was no further change of magnetization monitored at 5 K in 5000 Oe. The magnetization before and after irradiation was measured at 5 K in a field range of 0–50,000 Oe. The plot of the magnetization versus the magnetic field was analyzed in terms of the Brillouin function.

### 3.6. Low-Temperature UV-Vis Measurements

Low-temperature UV-vis spectra were obtained by using a variable-temperature liquid-nitrogen cryostat (DN 1704, Oxford, Oxfordshire, UK) equipped with quartz windows. The diazo compound was dissolved in dry 2-MTHF (1.5 × 10^−3^ M), placed in a long-necked quartz cuvette of 1-mm path length, and degassed thoroughly by five freeze-degas-thaw cycles at a pressure near 10^–5^ Torr. The cuvette was flame-sealed under reduced pressure, placed in the cryostat, and cooled to 77 K. The sample was irradiated for several min in the spectrometer with a Wacom 500 W Xe lamp using a filter (λ > 350 nm), and the spectral changes were recorded at appropriate time intervals. The spectral changes upon thawing were also monitored by carefully controlling the matrix temperature with an Oxford Instrument Intelligent Temperature Controller (ITC 4).

### 3.7. Laser Flash Photolysis

Laser flash photolysis measurements were made on a TSP-601 flash spectrometer (Unisoku, Osaka, Japan). The excitation source for the laser flash photolysis was a LEXTRA XeCl excimer laser (Lambda Physik, Göttingen, Germany, output energy 100 mJ, wavelength 308 nm, pulse duration 10 ns). A 150-W xenon short arc lamp (L2195, Hamamatsu Photonics, Shizuoka, Japan) was used as the probe source, and the monitoring beam, guided using an optical fiber scope, was arranged in an orientation perpendicular to the excitation source. The probe beam was monitored with a Hamamatsu R2949 photomultiplier tube through a Hamamatsu S3701-512Q linear image sensor. Timing of the laser excitation pulse, the probe beam, and the detection system was achieved through a model DS-8631 digital synchroscope (Iwatsu, Tokyo, Japan) which was interfaced to a 9821 RA266 computer (NEC, Tokyo, Japan).

A sample was placed in a long-necked Pyrex tube that had a sidearm connected to a quartz cuvette and was degassed by five freeze-degas-thaw cycles at a pressure near 10^−5^ Torr immediately prior to being photolyzed. The sample system was sealed, and the solution was transferred to the quartz cuvette, which was placed in the sample chamber of the flash photolysis apparatus.

## 4. Conclusions

A new diphenyldiazomethane **1a**-N_2_ with four iodine groups at all *ortho* positions was synthesized, and the triplet carbene, ^3^**1a**, generated by its photolysis, was characterized. In low-temperature matrices, an unusual stability was revealed from the time course of the absorption bands. In benzene at room temperature, ^3^**1a** decayed very slowly according to a second-order process to afford the corresponding phenanthrene. The present results demonstrated that carbene ^3^**1a** is one of the unusually persistent triplet carbenes with a half-life of over 10 min, and the *ortho*-iodine group is a simple and effective kinetic protector for triplet diphenylcarbene. This strategy will pave a road to the spin source of organic magnetic materials.

## Figures and Tables

**Table 1:** Kinetic Data of Carbenes **1**.

Carbenes	*2k/*ε*l* (s^−1^)	*t*_1/2_ (s)	*k*_O2_ (M^−1^·s^−1^)	*k*_CHD_ (M^−1^·s^−1^)	*Kk*_MeOH_^1^ (M^−2^·s^−1^)
**1a**	0.0055 0.33 min^−1^	1.1 × 10^3^ 18.4 min	6.5 × 10^5^	1.5	0.033
**1b**	0.17	2.4 × 10	7.3 × 10^6^	3.9 × 10	0.30
**1c**	0.35	1.6 × 10	2.1 × 10^7^	5.3 × 10^2^	0.42

^1^ *K* = *k*_TS_/*k*_ST_.

## Supplementary Materials

Supplementary materials can be accessed at: http://www.mdpi.com/1420-3049/21/11/1545/s1.
