# Supplementary material for: Diphenylcarbene Protected by Four ortho-Iodine Groups: An Unusually Persistent Triplet Carbene
Source: Molecules. 2016 Nov 15;21(11):1545. doi: 10.3390/molecules21111545 (PMC6272868; doi:10.3390/molecules21111545)
Supplement: Supplementary file 1 [file molecules-21-01545-s001.pdf]

Supplementary Materials: Diphenylcarbene Protected by Four *ortho*-Iodine Groups: An Unusually Persistent Triplet Carbene

Katsuyuki Hirai, Kana Bessho, Kosaku Tsujita and Toshikazu Kitagawa

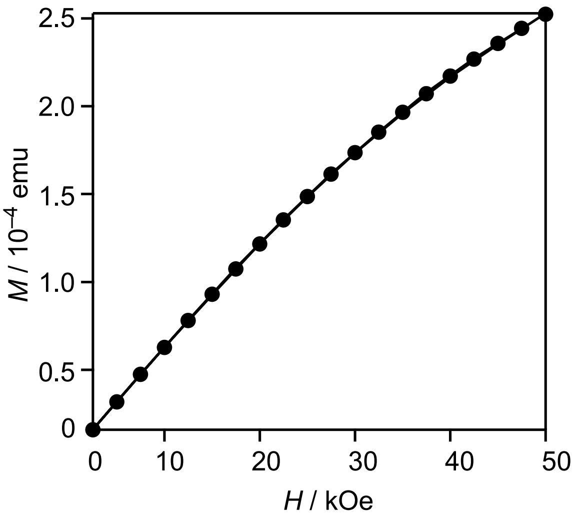

Figure S1. A plot of *M* vs. *H* of the photoproduct from **1a**-N_2 measured at 5.0 K. The solid line represents a theoretical curve with *S* = 0.93.

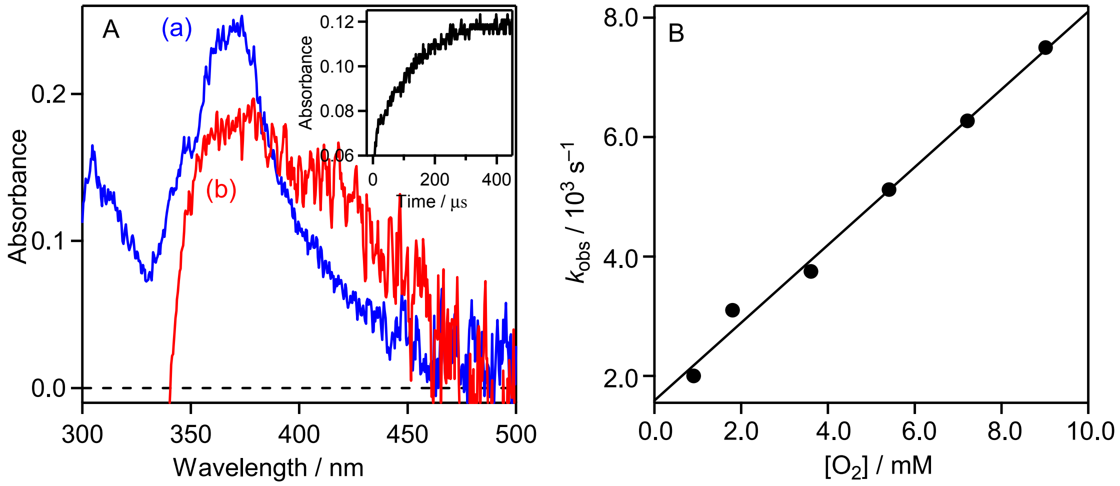

Figure S2. Laser flash photolysis (λ = 308 nm) of **1a**-N_2 in benzene at 25 °C: (**A**) Transient absorption spectra obtained (**a**) in degassed benzene and (**b**) in O_2 saturated benzene recorded after 100 μs. The inset shows the time course of the absorption monitored at 415 nm; (**B**) A plot of the growth rate constant of the diaryl ketone oxide **1a**-O_2 monitored at 415 nm as a function of the oxygen concentration.

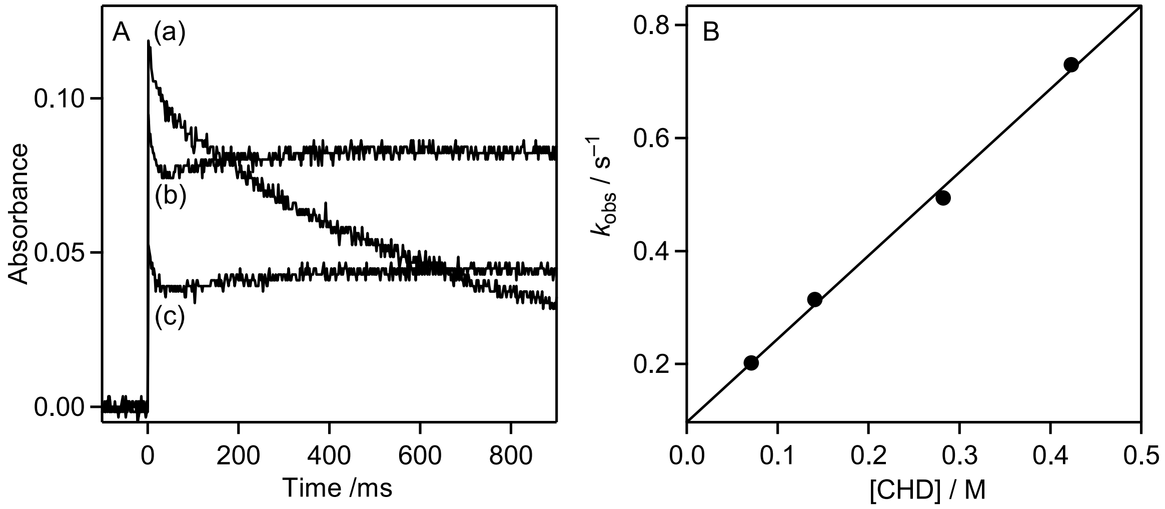

Figure S3. Laser flash photolysis (λ = 308 nm) of **1a**-N_2 in degassed benzene containing 1,4-cyclohexadiene: (**A**) Time courses of the absorbance obtained in the presence of 1,4-cyclohexadiene (0.265 M) recorded at 350 nm (**a**), 410 nm (**b**), and 430 nm (**c**); (**B**) A plot of the decay rate constant of carbene **1a** monitored at 350 nm as a function of the 1,4-cyclohexadiene concentration.

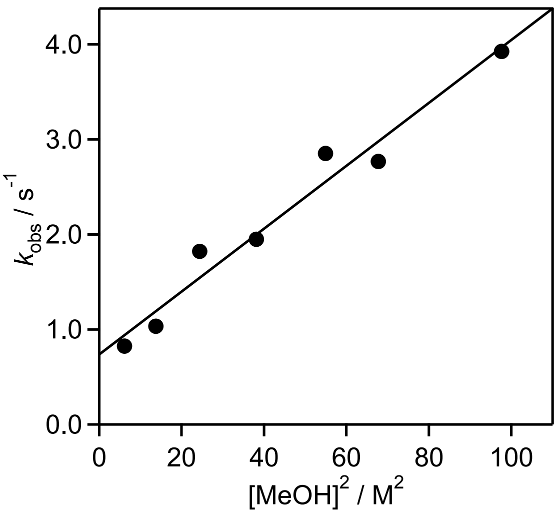

Figure S4. A plot of the decay rate constant of carbene **1a** monitored at 366 nm as a function of the square of the methanol concentration.

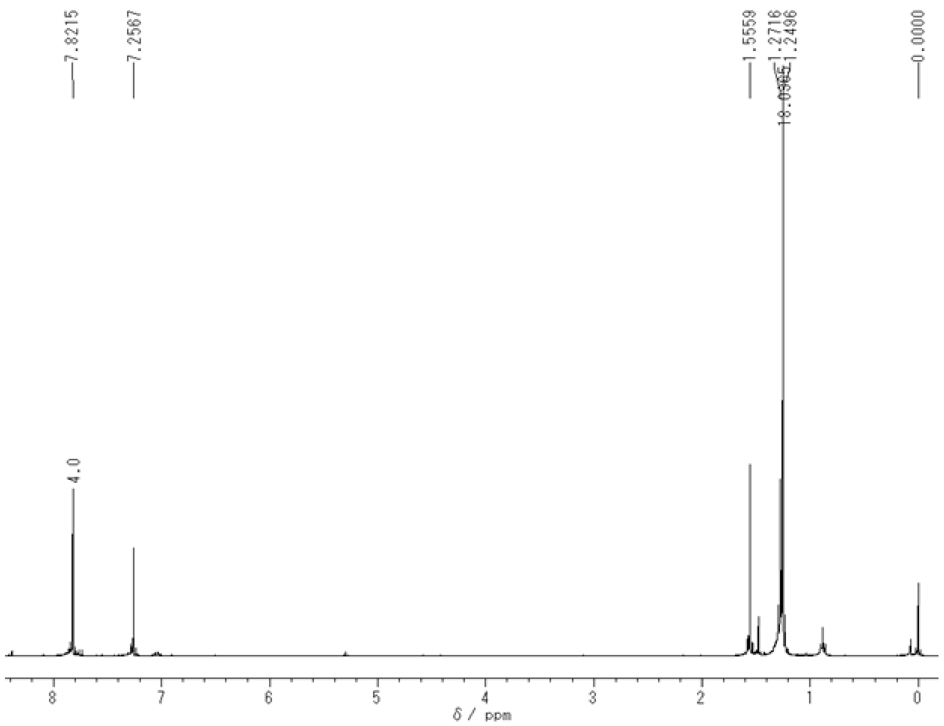

Figure S5. ^{1}H-NMR spectrum of 5-*tert*-butyl-1,2,3-triiodobenzene (**3**) (300 MHz, $CDCl_3$).

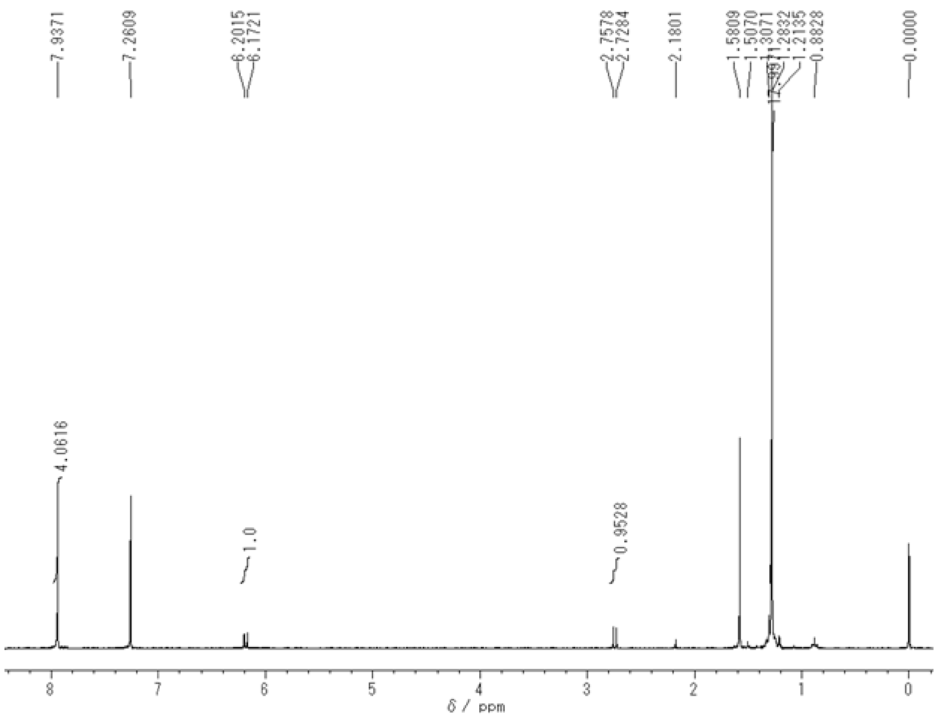

Figure S6. ^{1}H-NMR spectrum of bis(4-*tert*-butyl-2,6-diiodophenyl)methanol (**4**) (300 MHz, $CDCl_3$).

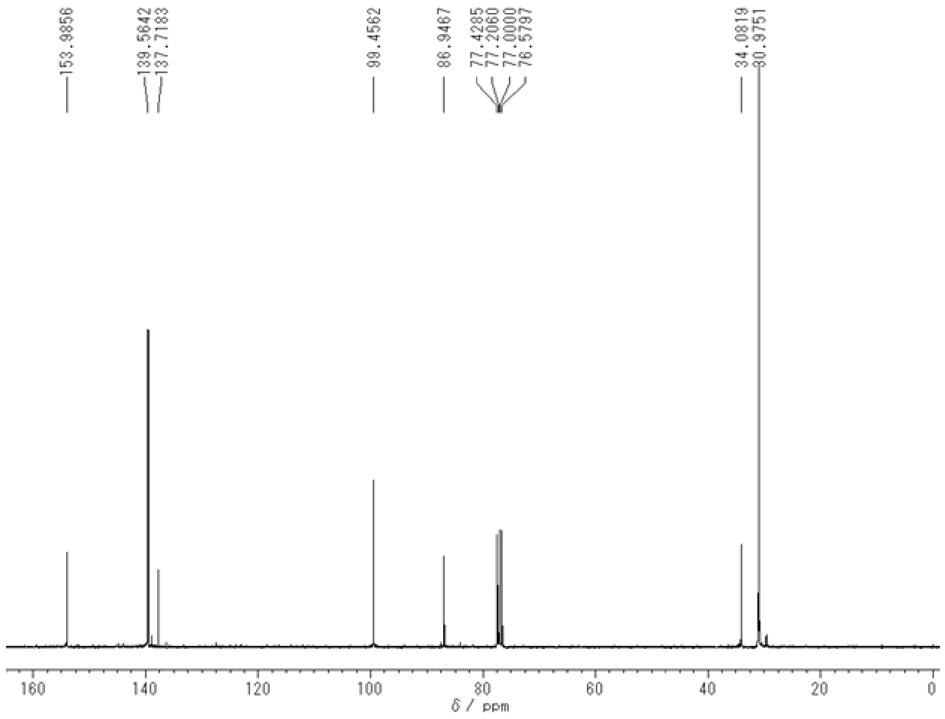

Figure S7. ^{13}C-NMR spectrum of bis(4-*tert*-butyl-2,6-diiodophenyl)methanol (**4**) (75.5 MHz, $CDCl_3$).

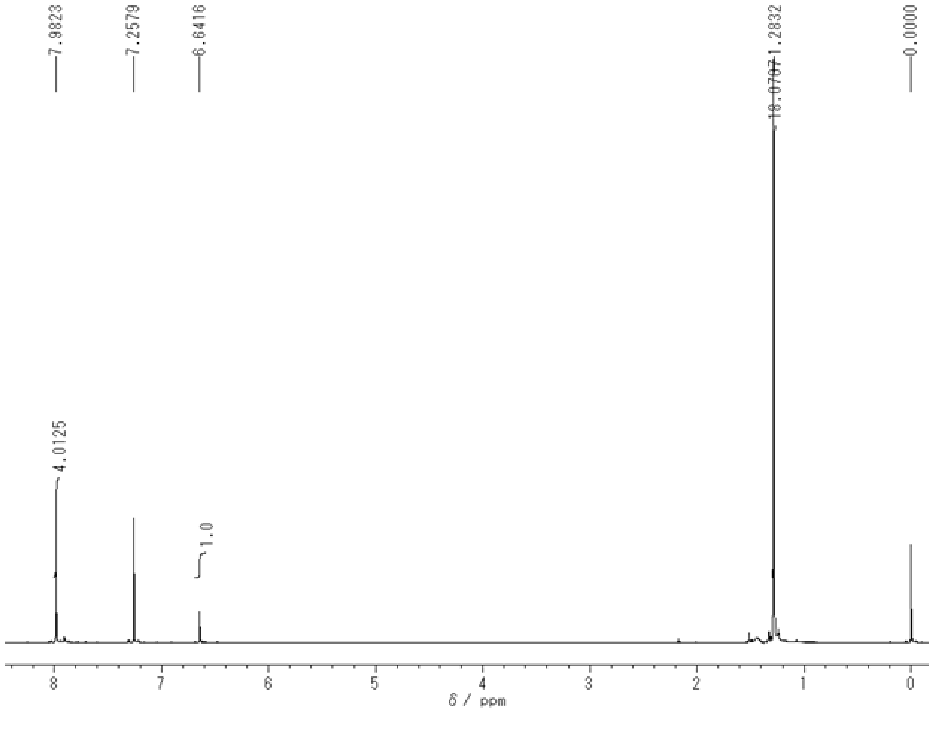

Figure S8. ^{1}H-NMR spectrum of bis(4-*tert*-butyl-2,6-diiodophenyl) chloromethane (**5**) (300 MHz, $CDCl_3$).

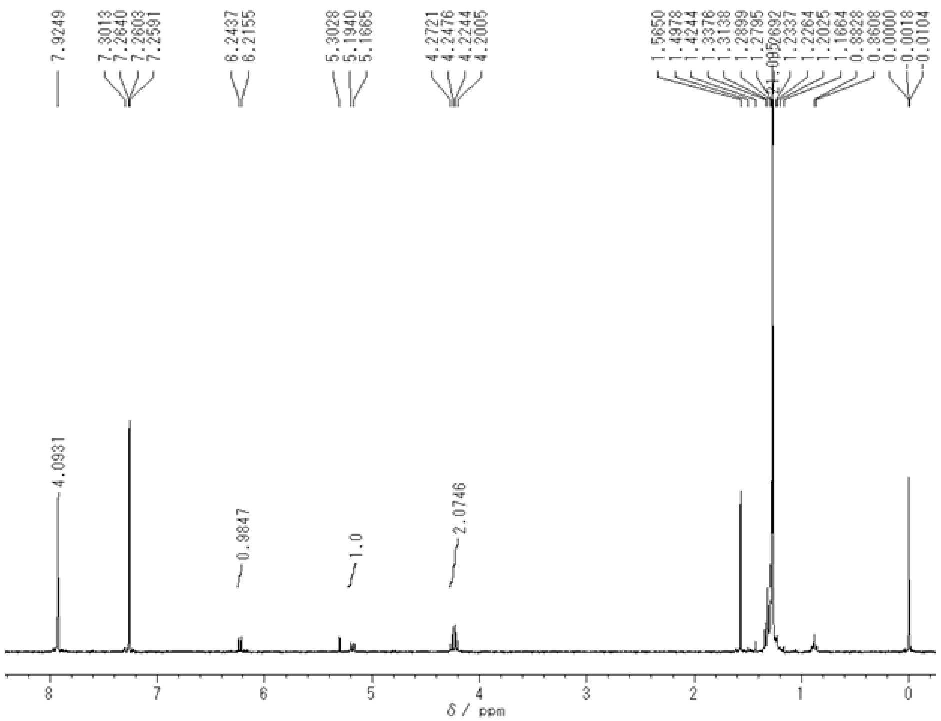

Figure S9. ^{1}H-NMR spectrum of ethyl *N*-[bis(4-*tert*-butyl-2,6-diiodophenyl) methyl]carbamate (**6**) (300 MHz, $CDCl_3$).

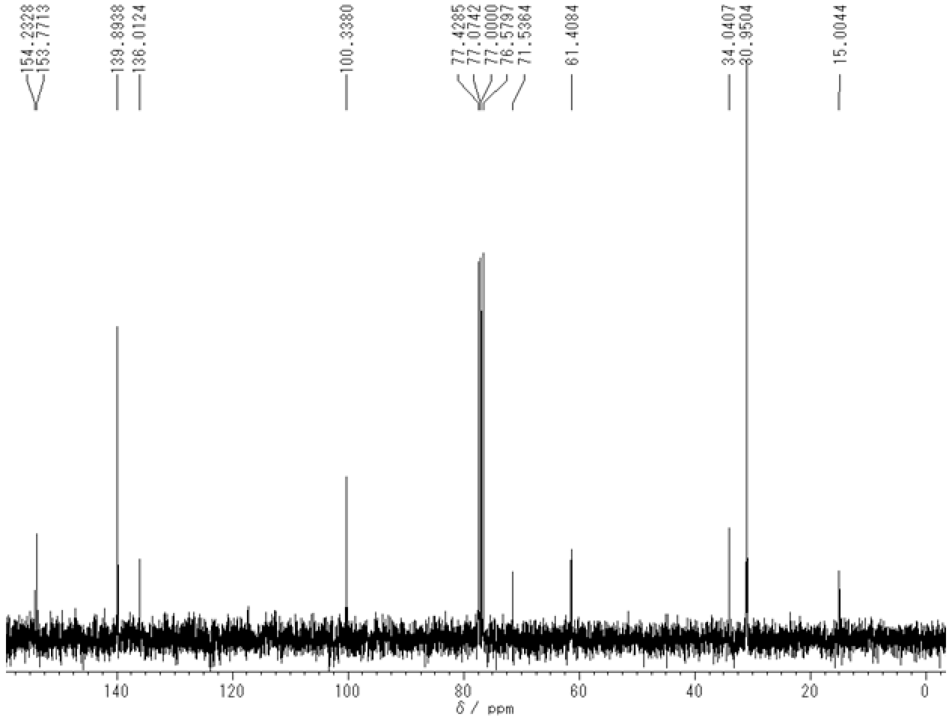

Figure S10. ^{13}C-NMR spectrum of ethyl *N*-[bis(4-*tert*-butyl-2,6-diiodophenyl) methyl]carbamate (**6**) (75.5 MHz, $CDCl_3$).

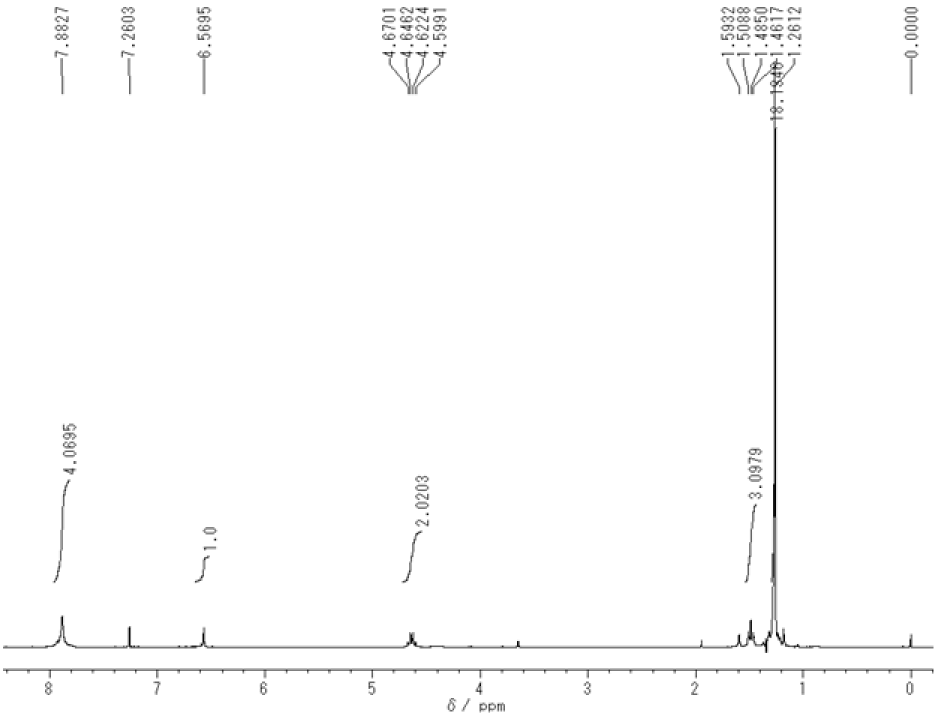

Figure S11. ^{1}H-NMR spectrum of ethyl *N*-nitroso-*N*-[bis(4-*tert*-butyl-2,6-diiodophenyl)methyl]carbamate (**7**) (300 MHz, $CDCl_3$).

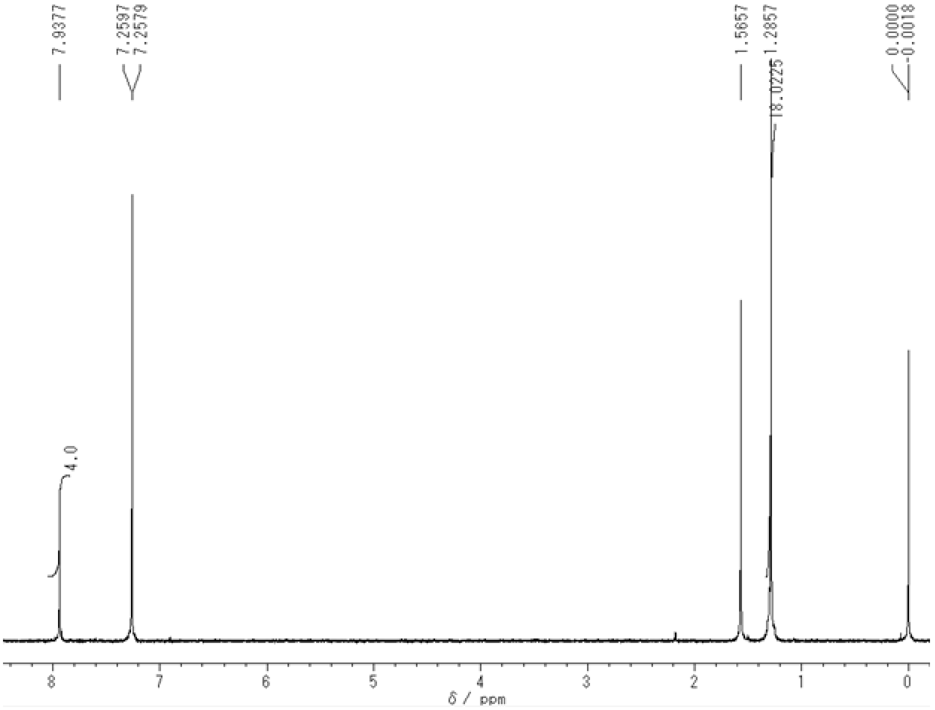

Figure S12. ^{1}H-NMR spectrum of bis(4-*tert*-butyl-2,6-diiodophenyl)diazomethane (**1a**-N_2) (300 MHz, $CDCl_3$).

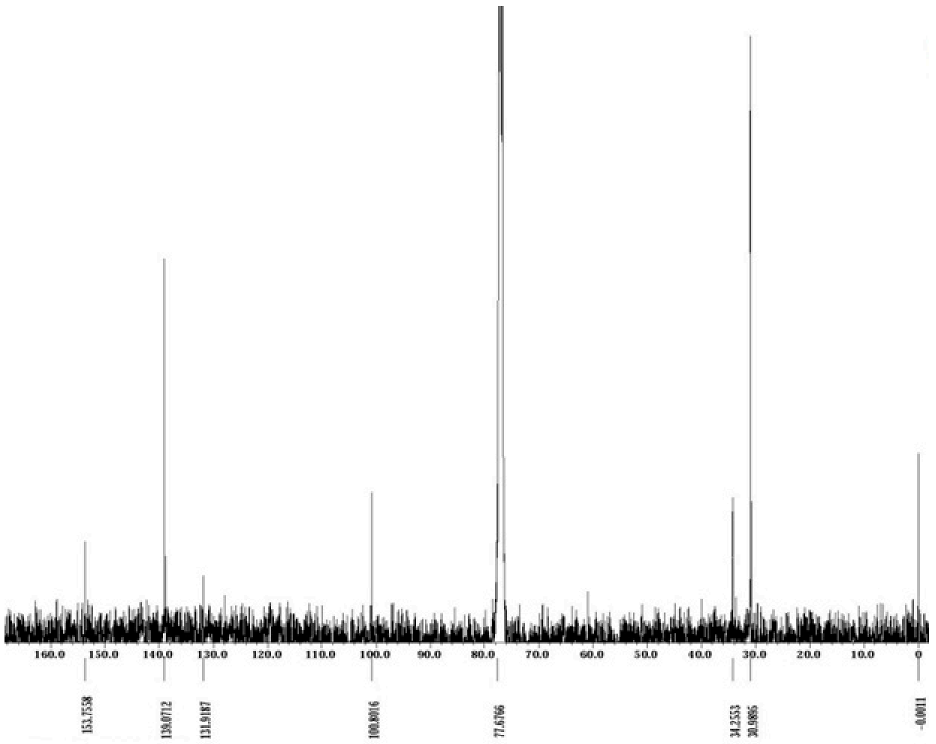

Figure S13. ^{13}C-NMR spectrum of bis(4-*tert*-butyl-2,6-diiodophenyl) diazomethane (**1a**-N_2) (100 MHz, $CDCl_3$).

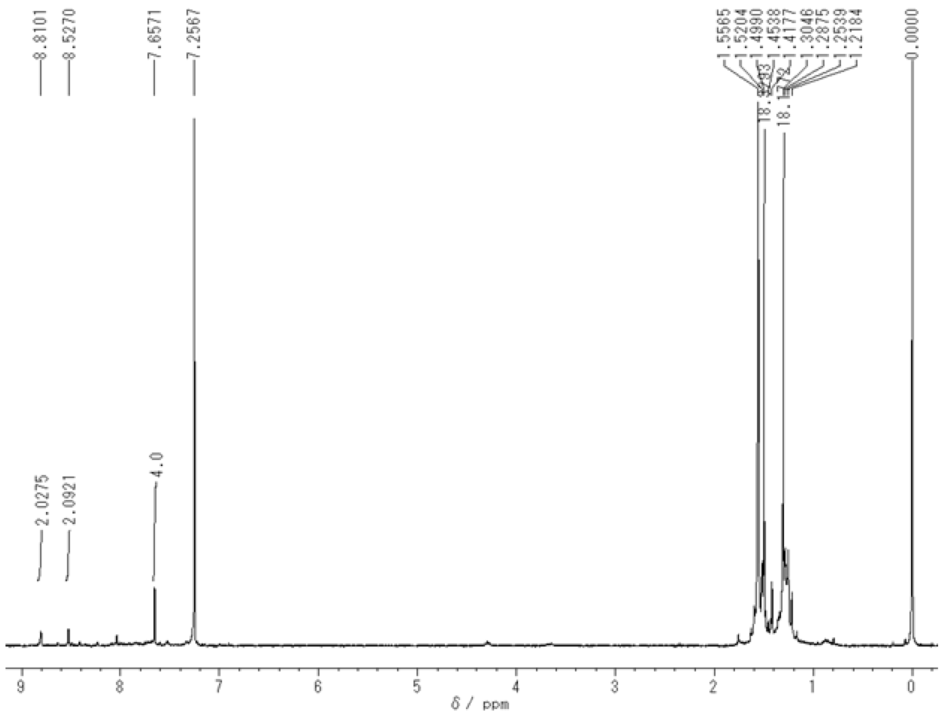

Figure S14. ^{1}H-NMR spectrum of 3,6-di-*tert*-butyl-9,10-bis(4-*tert*-butyl-2,6-diiodophenyl)-1,8-diiodophenanthrene (**2a**) (300 MHz, $CDCl_3$).

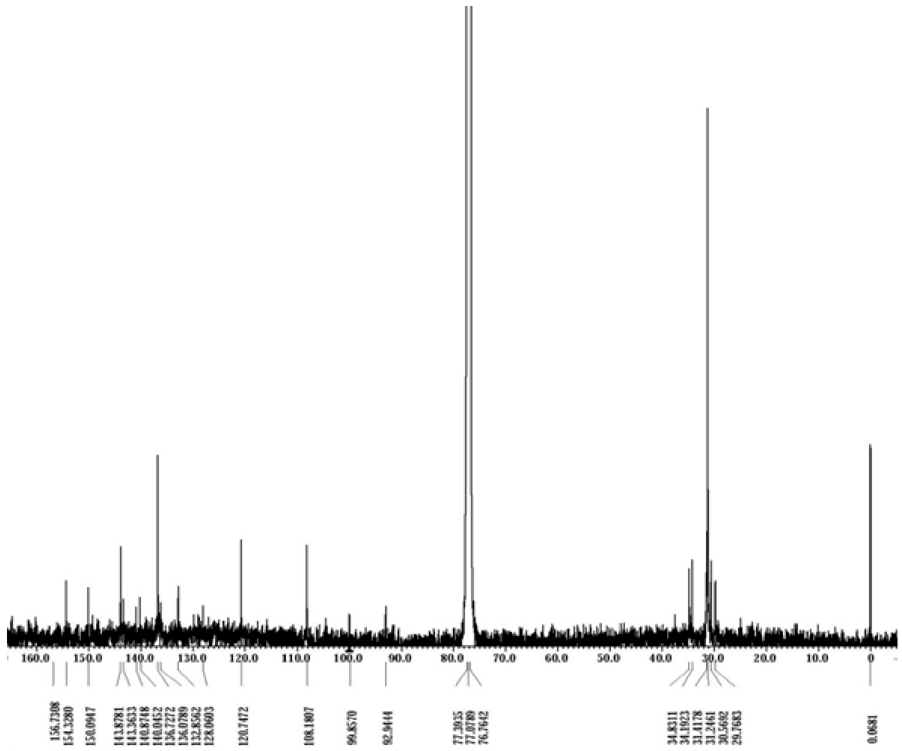

Figure S15. ^{13}C-NMR spectrum of 3,6-di-*tert*-butyl-9,10-bis(4-*tert*-butyl-2,6-diiodophenyl)-1,8-diiodophenanthrene (**2a**) (100 MHz, $CDCl_3$).

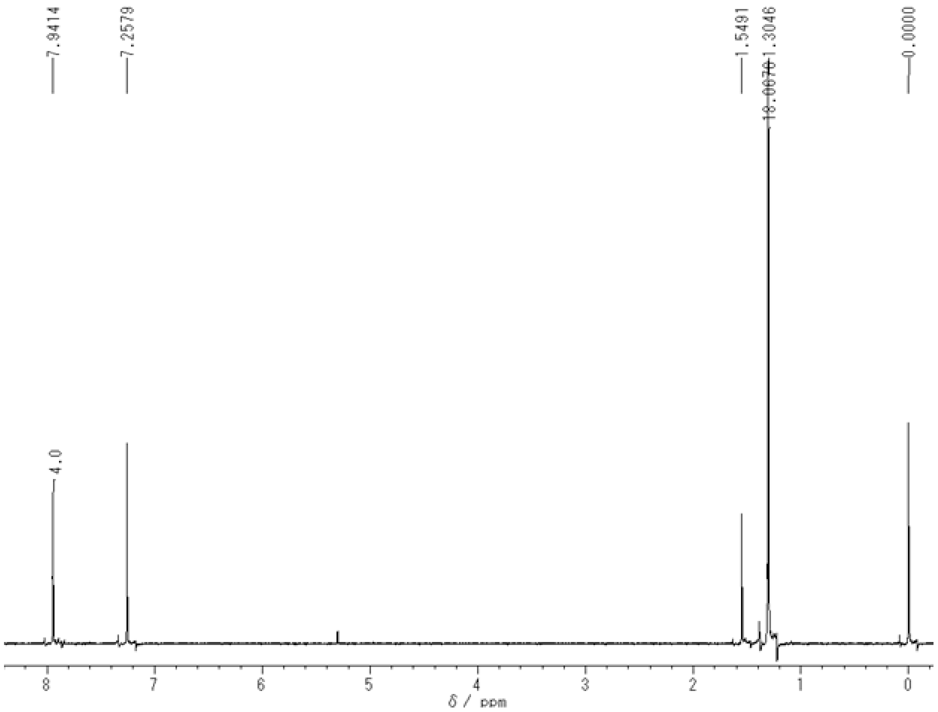

Figure S16. ^{1}H-NMR spectrum of bis(4-*tert*-butyl-2,6-diiodophenyl) ketone (**1a**-O) (300 MHz, $CDCl_3$).

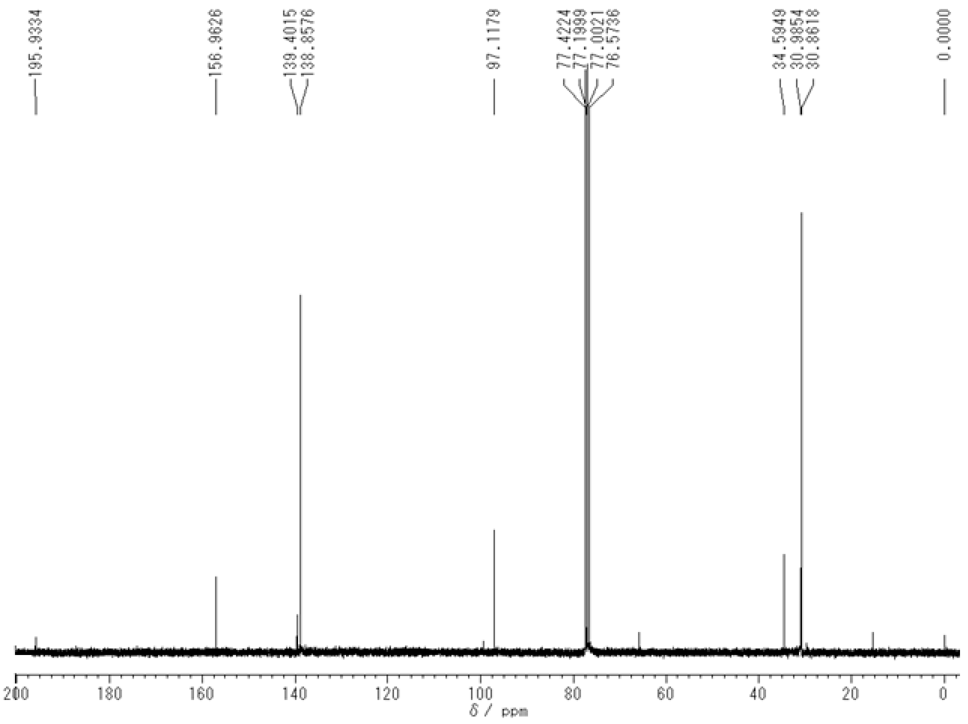

Figure S17. ^{13}C-NMR spectrum of bis(4-*tert*-butyl-2,6-diiodophenyl) ketone (**1a**-O) (75.5 MHz, $CDCl_3$).

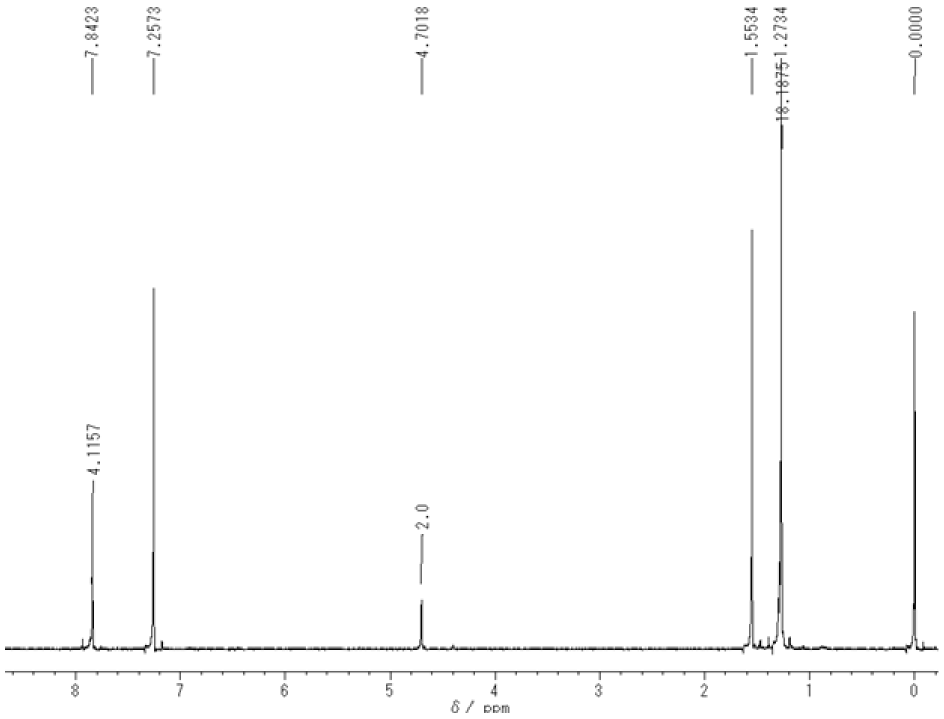

Figure S18. ^{1}H-NMR spectrum of bis(4-*tert*-butyl-2,6-diiodophenyl)methane (**1a**-H_2) (300 MHz, $CDCl_3$).

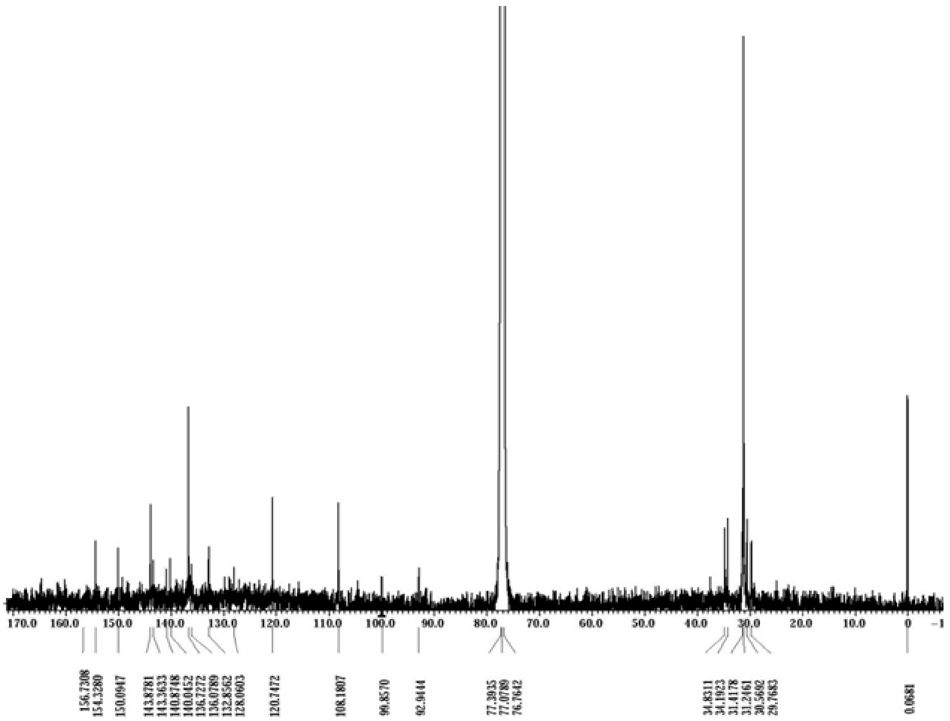

Figure S19. ^{13}C-NMR spectrum of bis(4-*tert*-butyl-2,6-diiodophenyl)methane (**1a**-H_2) (300 MHz, $CDCl_3$).

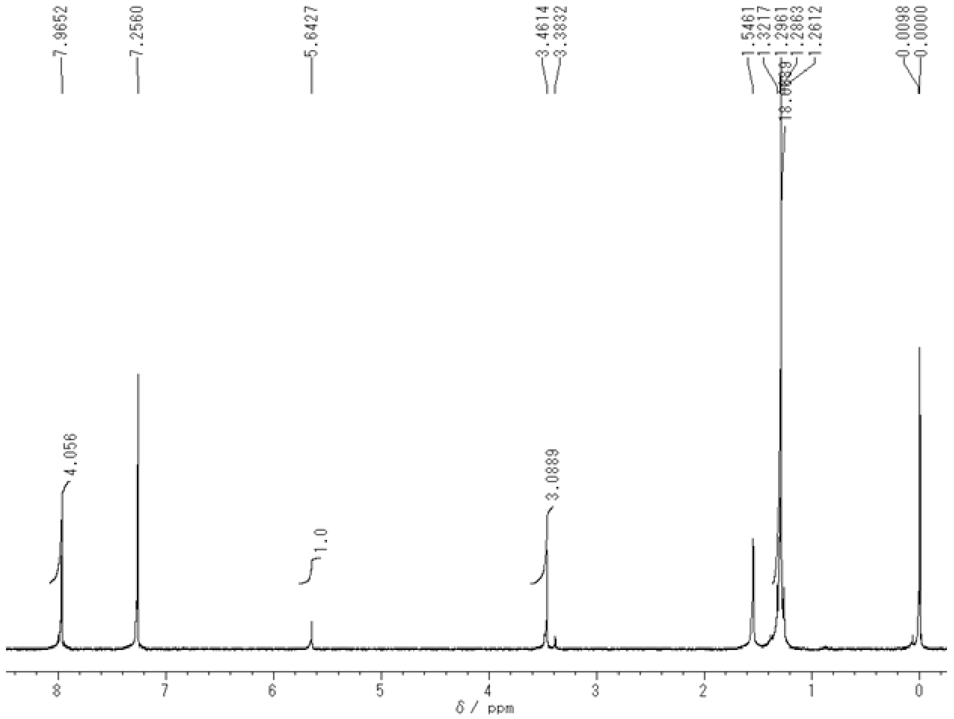

Figure S20. ^{1}H-NMR spectrum of bis(4-*tert*-butyl-2,6-diiodophenyl)methyl methyl ether (**1a**-HOMe) (300 MHz, $CDCl_3$).

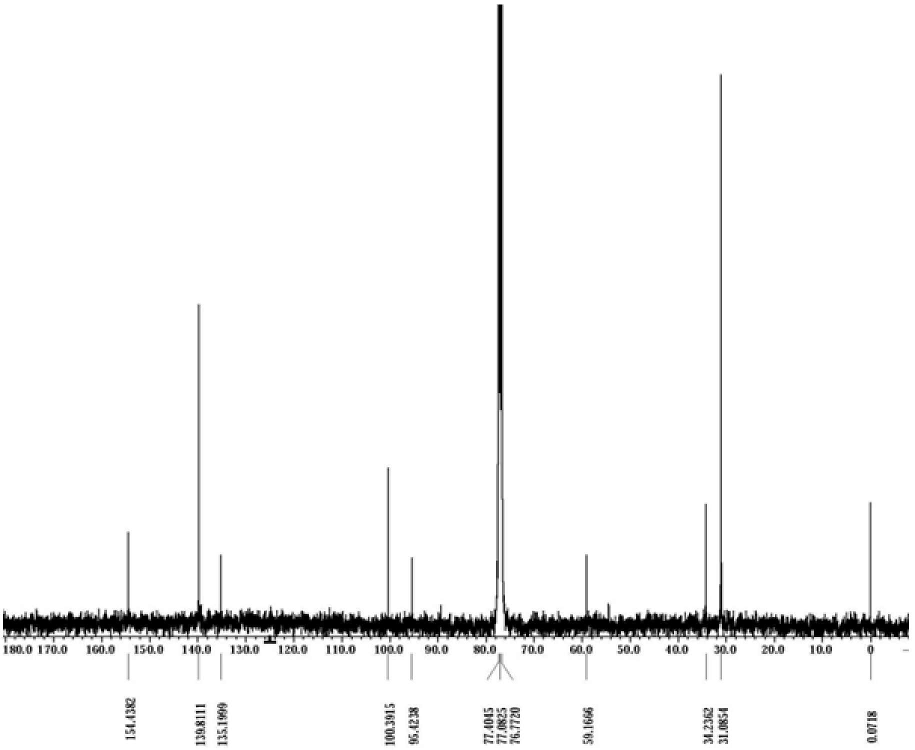

Figure S21. ^{13}C-NMR spectrum of bis(4-*tert*-butyl-2,6-diiodophenyl)methyl methyl ether (**1a**-HOMe) (300 MHz, $CDCl_3$).

Table S1. Cartesian Coordinates of the Optimized Structure of [1]**1a**.

Atomic Coordinates (Angstroms)			
**Type**	**X**	**Y**	**Z**
C	−3.6177457716	0.5763654154	0.7295425607
C	−4.0146196452	0.6853191398	−0.6141400091
C	−3.0023296681	0.8466749953	−1.5651168247
C	−1.6532834881	0.8596182984	−1.2203702753
C	−1.2417394443	0.7951955107	0.1390283296
C	−2.2908007036	0.6768979818	1.0960621290
C	0.0704288587	0.9907152751	0.6173272625
C	1.2672875330	0.3278993430	0.2840235201
C	1.4387953154	−1.0822664935	0.1123938811
C	2.7054442536	−1.6389918690	0.0105158615
C	3.8699750853	−0.8567346114	−0.0346207121
C	3.7158844028	0.5313818482	0.0636696229
C	2.4683616363	1.0981252248	0.2652272485
C	−5.5012654884	0.6406534630	−0.9771034449
C	−6.2244384863	1.8088980283	−0.2856273082
C	−6.1000581666	−0.6927112341	−0.4976114622
C	−5.7346191233	0.7558360419	−2.4862387789
C	5.2350631936	−1.5312249431	−0.1866274827
C	6.3845453365	−0.5196284595	−0.1889044612
C	5.2660490377	−2.3042567429	−1.5166282337
C	5.4516410656	−2.5058754336	0.9832936503
I	2.3057423254	3.1838695817	0.4435958228
I	−0.1769891919	−2.4317217501	0.0810148766
I	−0.2697723325	1.0743873556	−2.7920289647
I	−1.7301039640	0.5427110297	3.1172203754
H	−4.3617921788	0.4403314908	1.5057748741
H	−3.2581268781	0.9390606128	−2.6124143568
H	2.7881151983	−2.7172235836	−0.0650116924
H	4.5783889779	1.1825615681	0.0161973699
H	−7.2923428802	1.7909022447	−0.5266854161
H	−5.8188893946	2.7701526313	−0.6150236026
H	−6.1302381642	1.7641591824	0.8026648769
H	−7.1648233812	−0.7418898869	−0.7477505056
H	−6.0095154421	−0.8171797688	0.5847171849
H	−5.6006432465	−1.5415519767	−0.9744578680
H	−6.8074160428	0.7227747921	−2.6964936255
H	−5.2689420701	−0.0675194065	−3.0363479509
H	−5.3522263897	1.6983175466	−2.8895076320
H	7.3374055854	−1.0466555215	−0.2892717011
H	6.4244104934	0.0556814675	0.7411751535
H	6.3116516482	0.1827158462	−1.0250490774
H	6.2356090500	−2.7958427302	−1.6449615334
H	5.1129923612	−1.6310837080	−2.3655958162
H	4.4941569828	−3.0773750211	−1.5611049469
H	6.4279977069	−2.9924508230	0.8929941076
H	4.6923790709	−3.2919223385	1.0093196490
H	5.4224324237	−1.9802996132	1.9423423256

Electronic Energy = −28372.8365588/Hartree; Zero Point Energy = 0.371891/Hartree; Gibbs Free Energy = −28372.464668/Hartree.

Table S2. Cartesian Coordinates of the Optimized Structure of ^{3}**1a**.

Atomic Coordinates (Angstroms)			
**Type**	**X**	**Y**	**Z**
C	−2.7587580913	−0.1425870102	2.5225297874
C	−3.8803761940	−0.0196499334	1.6804595432
C	−3.6376105817	0.1085155941	0.3057148498
C	−2.3516632021	0.1151945892	−0.2129599605
C	−1.2007125601	−0.0089415347	0.6270423836
C	−1.4683381748	−0.1390821523	2.0292673604
C	0.0770316306	−0.0037899517	0.1249271352
C	1.3539713896	0.0017795186	−0.3792386486
C	2.0202172214	−1.2016858695	−0.7824003297
C	3.3058778939	−1.1826187239	−1.2875758285
C	4.0325081309	0.0141992344	−1.4353608362
C	3.3963467235	1.2013946483	−1.0464730240
C	2.1069535958	1.2075900430	−0.5365184664
C	−5.2894812457	−0.0289429123	2.2791044638
C	−5.4270502665	1.1440528788	3.2641992048
C	−5.5140424753	−1.3563990712	3.0225346003
C	−6.3747489892	0.1121423681	1.2082290566
C	5.4550692855	−0.0210015198	−2.0002136769
C	6.0786780186	1.3747419750	−2.0866778537
C	5.4222956526	−0.6240347089	−3.4146373536
C	6.3385696269	−0.8895958163	−1.0890440681
I	1.2305637269	3.0316283948	0.0212985167
I	1.0069303054	−3.0312719572	−0.5955801292
I	−2.0805394442	0.3124401327	−2.2850923914
I	0.1488794730	−0.3280191268	3.3551634707
H	−2.8998653321	−0.2436526084	3.5923089851
H	−4.4660678667	0.2059272067	−0.3829493875
H	3.7588959466	−2.1240271839	−1.5758481585
H	3.9124503026	2.1473759599	−1.1405228510
H	−6.4279603699	1.1532482122	3.7074118023
H	−5.2723236926	2.1014945682	2.7577002909
H	−4.7042141720	1.0792511329	4.0820301544
H	−6.5162619061	−1.3813505631	3.4621702457
H	−4.7943421565	−1.4991268918	3.8331985728
H	−5.4219421111	−2.2076598015	2.3413472501
H	−7.3612282961	0.0997940958	1.6801225557
H	−6.3475736992	−0.7105224525	0.4870433619
H	−6.2878052102	1.0545407141	0.6587500363
H	7.0904581374	1.3010941764	−2.4951952807
H	6.1574236495	1.8496886598	−1.1039307780
H	5.5085886449	2.0377745236	−2.7447102315
H	6.4321181427	−0.6617747890	−3.8355382165
H	4.7992333671	−0.0221673004	−4.0829891193
H	5.0253943276	−1.6428484303	−3.4167804897
H	7.3608351912	−0.9317620974	−1.4783073290
H	5.9694580599	−1.9163853970	−1.0182427893
H	6.3789585934	−0.4793388231	−0.0754984297
C	−2.7913540064	−0.2126258212	2.5957931654
C	−3.8682882486	−0.0303410842	1.7032375166

C	−3.5607153009	0.0785858940	0.3374490670
C	−2.2548785866	0.0079150477	−0.1247461335
C	−1.1401850518	−0.1788295173	0.7603408836
C	−1.4863571644	−0.2847225098	2.1523527933
C	0.1585783143	−0.2804233236	0.3293358610
C	1.3970988456	−0.1788892109	−0.2525502527
C	2.1213847521	−1.3388349901	−0.6982988883
C	3.3794261808	−1.2527576686	−1.2593261187
C	4.0321758781	−0.0138102189	−1.4284691633
C	3.3483494382	1.1369831069	−1.0043239908
C	2.0839363982	1.0679254085	−0.4383397490
C	−5.3055977620	0.0368835397	2.2439205589
C	−5.4270542211	1.2221162219	3.2323545957
C	−5.6370617849	−1.2828585699	2.9822161231
C	−6.3434019270	0.2354964997	1.1227989281
C	5.4363348548	0.0309545094	−2.0522105298
C	5.9863657457	1.4665407255	−2.1519923745
C	5.3834182389	−0.5680726065	−3.4787132016
C	6.4097386483	−0.8010581866	−1.1822082123
I	1.1493443793	2.8696265182	0.1721286907
I	1.2105150441	−3.2403499686	−0.4756510214
I	−1.9090531582	0.1777534285	−2.2089234335
I	0.0753215387	−0.5639340271	3.5588359480
H	−2.9829335272	−0.2996428927	3.6590353281
H	−4.3526482643	0.2208883071	−0.3858379611
H	3.8698899997	−2.1664516176	−1.5745464979
H	3.8072576169	2.1105303866	−1.1145983877
H	−6.4466328172	1.2773277450	3.6325361012
H	−5.2047639675	2.1728180900	2.7347823162
H	−4.7422842024	1.1195149596	4.0806917327
H	−6.6582255384	−1.2467717672	3.3805387606
H	−4.9587116205	−1.4632411121	3.8226591344
H	−5.5663292044	−2.1405733992	2.3039112907
H	−7.3482459257	0.2754538201	1.5576261223
H	−6.3296809802	−0.5894075788	0.4012729285
H	−6.1832369625	1.1725543186	0.5773795031
H	6.9859802767	1.4447801077	−2.5999682272
H	6.0772194318	1.9395768318	−1.1674956707
H	5.3565553892	2.1037810635	−2.7832447562
H	6.3812600128	−0.5498006853	−3.9333658939
H	4.7061974279	0.0061720098	−4.1209263119
H	5.0396111264	−1.6077265915	−3.4736341437
H	7.4154590454	−0.7844465097	−1.6192325652
H	6.0978090585	−1.8479248869	−1.1059457320
H	6.4732135797	−0.3950517952	−0.1663801323

Electronic Energy = −28372.8674296/Hartree; Zero Point Energy = 0.3716611/Hartree; Gibbs Free Energy = −28372.495769/Hartree.

Table S3. Cartesian Coordinates of the Optimized Structure of [1]**1b**.

Atomic Coordinates (Angstroms)			
**Type**	**X**	**Y**	**Z**
C	−3.6681736398	−0.0340270207	0.8200401776
C	−4.0504161448	0.0251900975	−0.5322183146
C	−3.0354313845	0.2001908144	−1.4771576960
C	−1.6977148964	0.2747423245	−1.1059876652
C	−1.2882904721	0.2859086102	0.2568202790
C	−2.3528911286	0.1348930480	1.1955136353
C	−0.0026085301	0.6274790959	0.7155117016
C	1.2654365493	0.1516790904	0.3413020014
C	1.6448553975	−1.2214660709	0.2180342260
C	2.9777486279	−1.5826908045	0.0847596236
C	4.0054810890	−0.6360661807	−0.0470065518
C	3.6411157499	0.7159100822	−0.0064033922
C	2.3300671502	1.0944453826	0.2281904194
C	−5.5282135842	−0.0964693992	−0.9114367326
C	−6.3122446455	1.0475653647	−0.2460930960
C	−6.0674416198	−1.4491811164	−0.4164020885
C	−5.7481610935	−0.0151957613	−2.4246425923
C	5.4521472744	−1.1037667129	−0.2231578688
C	6.4282162915	0.0683453046	−0.3603860378
C	5.5527569585	−1.9662285487	−1.4927999920
C	5.8640817976	−1.9370711082	1.0023429021
I	1.8309237581	3.1318513413	0.3123633176
I	0.2480641289	−2.7933133027	0.3146002936
Br	−0.4321382646	0.4444919307	−2.4932482071
Br	−1.9025532088	0.0655947790	3.0213737155
H	−4.4092451070	−0.1880577196	1.5954010584
H	−3.2695523910	0.2514169020	−2.5318147245
H	3.2221583477	−2.6383007928	0.0513888679
H	4.3899277981	1.4881885905	−0.1258778097
H	−7.3749117193	0.9751326720	−0.4985522315
H	−5.9485015831	2.0212623415	−0.5876243291
H	−6.2289820989	1.0243510367	0.8437453785
H	−7.1264761080	−1.5513929614	−0.6735025237
H	−5.9798454824	−1.5551184811	0.6681755668
H	−5.5253717272	−2.2803299781	−0.8777934251
H	−6.8149715041	−0.1062679728	−2.6471426372
H	−5.2331645745	−0.8213440225	−2.9561811522
H	−5.4093721289	0.9403553380	−2.8366061277
H	7.4460376975	−0.3128423657	−0.4821223241
H	6.4242852656	0.7112570003	0.5252670644
H	6.2041077323	0.6861726311	−1.2353896670
H	6.5812543262	−2.3138236676	−1.6324881074
H	5.2656912602	−1.3935428568	−2.3799697160
H	4.9096821327	−2.8492221615	−1.4441417298
H	6.8944601228	−2.2896403087	0.8917562376
H	5.2262204007	−2.8148769414	1.1335947629
H	5.8061051805	−1.3420885219	1.9187025108

Electronic Energy = −19735.3876278/Hartree; Zero Point Energy = 0.372335/Hartree; Gibbs Free Energy = −19735.015293/Hartree.

Table S4. Cartesian Coordinates of the Optimized Structure of [3]**1b**.

Atomic Coordinates (Angstroms)			
**Type**	**X**	**Y**	**Z**
C	−3.6828856226	0.4034644477	0.9493042025
C	−4.3039916066	−0.1577008631	−0.1830214562
C	−3.4689826840	−0.6507971002	−1.1961581388
C	−2.0891266248	−0.5887081954	−1.0907514840
C	−1.4307138994	−0.0238057459	0.0452712084
C	−2.3095782781	0.4683555507	1.0620386764
C	−0.0633863985	0.0413339499	0.1625445489
C	1.3067216517	0.0383297878	0.0481611227
C	2.1053983018	−1.0646131549	0.4868283115
C	3.4879035697	−1.0553758502	0.3771703059
C	4.1781391133	0.0371963059	−0.1668252564
C	3.4087745052	1.1339198662	−0.5993885302
C	2.0306656232	1.1477659641	−0.4997211457
C	−5.8317091799	−0.2122875085	−0.2647340111
C	−6.3947216799	1.2158963407	−0.1738616475
C	−6.3701284374	−1.0549209368	0.9040518319
C	−6.3186929332	−0.8389172228	−1.5743700358
C	5.7033744175	0.0777253840	−0.2974670854
C	6.3647824921	−1.1995737682	0.2281804092
C	6.2479390478	1.2695668185	0.5079085395
C	6.0808673281	0.2397371768	−1.7795218276
I	0.9646998303	2.8331656883	−1.1583975934
I	1.1463559504	−2.7393874358	1.3136030670
Br	−1.0383803433	−1.2771295475	−2.4903565748
Br	−1.5435394576	1.2245900646	2.6048673783
H	−4.2823315820	0.7983245376	1.7608935974
H	−3.8887252043	−1.0958482802	−2.0885714225
H	4.0345435148	−1.9231547881	0.7223202576
H	3.9031445623	1.9994034585	−1.0257540621
H	−7.4878381582	1.1962497566	−0.2280135580
H	−6.0263034275	1.8361557594	−0.9964401208
H	−6.1192282348	1.7074707268	0.7629541367
H	−7.4627289535	−1.1087154302	0.8614301330
H	−6.0979510758	−0.6321812212	1.8749407002
H	−5.9791128598	−2.0762395307	0.8641249320
H	−7.4120295055	−0.8546715384	−1.5918162580
H	−5.9757653102	−1.8721138364	−1.6851445373
H	−5.9860227814	−0.2703575593	−2.4480818174
H	7.4501526350	−1.1234407780	0.1178151889
H	6.0417932629	−2.0856836348	−0.3267845414
H	6.1556019249	−1.3630541999	1.2897654077
H	7.3381683099	1.3192161203	0.4219067784
H	5.9959245703	1.1743301670	1.5683234114
H	5.8458939566	2.2227909517	0.1544208244
H	7.1691231506	0.2792118436	−1.8921175519
H	5.6715498797	1.1569972864	−2.2112151693
H	5.7099176400	−0.6014708269	−2.3726911443

Electronic Energy = −19735.4181061/Hartree; Zero Point Energy = 0.371699/Hartree; Gibbs Free Energy = −19735.046407/Hartree.

Table S5. Cartesian Coordinates of the Optimized Structure of [1]**1c**.

Atomic Coordinates (Angstroms)			
**Type**	**X**	**Y**	**Z**
C	−3.6808041952	−0.0873935236	0.7809526357
C	−4.0459768644	0.0798490915	−0.5670458844
C	−3.0271314768	0.3849994010	−1.4736699790
C	−1.7007481091	0.4932979946	−1.0702492613
C	−1.3197763343	0.4054957299	0.2936991174
C	−2.3804379479	0.1067056513	1.1925032177
C	−0.0498209438	0.7695782490	0.7917103816
C	1.2079332986	0.2823184483	0.3741954638
C	1.5685442788	−1.0933896456	0.3541471615
C	2.8810332866	−1.5022738489	0.1839381003
C	3.9132785130	−0.5889166766	−0.0756447946
C	3.5698769356	0.7684122612	−0.1337282758
C	2.2747633200	1.1817188937	0.1195242591
C	−5.5102726836	−0.0779059387	−0.9843486968
C	−6.3603340162	0.9641557749	−0.2376051642
C	−5.9921137612	−1.4925005521	−0.6200826566
C	−5.7101157936	0.1259164754	−2.4884616115
C	5.3434522982	−1.0940857228	−0.2855985090
C	6.3256886630	0.0473206637	−0.5628584448
C	5.3706823705	−2.0580715815	−1.4836407159
C	5.8072557614	−1.8327606491	0.9813429963
Br	1.8357772579	3.0047513083	−0.0090192953
Br	0.2667815640	−2.4195653990	0.6748595500
Br	−0.4129048723	0.8779999324	−2.3927119374
Br	−1.9145706352	−0.1624475329	2.9937920185
H	−4.4209463223	−0.3577704918	1.5251963496
H	−3.2470100727	0.5217817016	−2.5242123791
H	3.0920586930	−2.5639976654	0.2347837751
H	4.3114430295	1.5228168902	−0.3616581255
H	−7.4136753925	0.8652131710	−0.5180304267
H	−6.0393061245	1.9808521212	−0.4821929171
H	−6.2952944759	0.8471698163	0.8472982295
H	−7.0375628889	−1.6236572094	−0.9162054358
H	−5.9281204084	−1.6858805621	0.4539014579
H	−5.3976396602	−2.2549832694	−1.1319483440
H	−6.7675353251	0.0050010062	−2.7392214506
H	−5.1492896481	−0.6045141313	−3.0791430772
H	−5.4119988471	1.1286759601	−2.8085293305
H	7.3316783349	−0.3582290516	−0.7010820890
H	6.3717398483	0.7584686787	0.2673350306
H	6.0684617297	0.5978227873	−1.4728195217
H	6.3878544919	−2.4266585319	−1.6495009492
H	5.0386612872	−1.5579007652	−2.3982161298
H	4.7269527782	−2.9275853700	−1.3274644732
H	6.8291643800	−2.2024686038	0.8513257803
H	5.1718135464	−2.6918595518	1.2113339093
H	5.7957111327	−1.1680037332	1.8500364417

Electronic Energy = −11099.1575441/Hartree; Zero Point Energy = 0.3728268/Hartree; Gibbs Free Energy = −11098.784717/Hartree.

Table S6. Cartesian Coordinates of the Optimized Structure of [3]**1c**.

Atomic Coordinates (Angstroms)			
**Type**	**X**	**Y**	**Z**
C	−2.7177258372	0.0299445491	2.4465608149
C	−3.8156544325	−0.0528319326	1.5694703263
C	−3.5387946432	−0.2329152249	0.2067874848
C	−2.2377672597	−0.3253912515	−0.2589578666
C	−1.1058334824	−0.2400358723	0.6063387580
C	−1.4195643446	−0.0598807248	1.9889226610
C	0.1865018384	−0.3322335184	0.1463698421
C	1.4144599933	−0.2256468628	−0.4627320214
C	2.0144165685	−1.3062845140	−1.1801388443
C	3.2477691829	−1.1953917323	−1.7878510572
C	3.9913558689	−0.0013707231	−1.7335543960
C	3.4295019077	1.0755951537	−1.0332178324
C	2.1923955372	0.9702819043	−0.4195545371
C	−5.2403519482	0.0544347649	2.1202994632
C	−5.4096082167	1.4119396533	2.8230116892
C	−5.4803811437	−1.0818030790	3.1284397240
C	−6.2957026192	−0.0510683932	1.0159313304
C	5.3554055197	0.0785496003	−2.4246812335
C	6.0053851347	1.4552989577	−2.2615557183
C	5.1776538018	−0.1974612281	−3.9271788821
C	6.2948414770	−0.9749417765	−1.8142594292
Br	1.5065298061	2.4448893496	0.5243704536
Br	1.0570650301	−2.9187798955	−1.3067921542
Br	−1.9363334841	−0.5903941718	−2.0960077350
Br	0.0069195783	0.0819539597	3.2045687340
H	−2.8742029129	0.1744537945	3.5089307092
H	−4.3420419421	−0.3060842419	−0.5145018033
H	3.6323149531	−2.0571420728	−2.3203075853
H	3.9557506671	2.0178217466	−0.9552158461
H	−6.4243187805	1.5092207052	3.2216108772
H	−5.2387093198	2.2390456529	2.1275582400
H	−4.7147882657	1.5311436979	3.6586194969
H	−6.4962794905	−1.0223039097	3.5314531081
H	−4.7875719127	−1.0357143893	3.9729551224
H	−5.3616469756	−2.0600175847	2.6532445292
H	−7.2951801919	0.0342836989	1.4510924821
H	−6.2476208312	−1.0117640482	0.4946347179
H	−6.1934995602	0.7472683957	0.2748102748
H	6.9722561359	1.4698504815	−2.7720041646
H	6.1873046544	1.6998396840	−1.2107930457
H	5.3940597925	2.2506209966	−2.6983531013
H	6.1428714688	−0.1416443825	−4.4402309144
H	4.5080751140	0.5363737673	−4.3855647183
H	4.7615150405	−1.1906872268	−4.1157523387
H	7.2770470111	−0.9307177896	−2.2952227010
H	5.9113129769	−1.9909054112	−1.9408877254
H	6.4346265358	−0.8028195561	−0.7429791880

Electronic Energy = −11099.1866364/Hartree; Zero Point Energy = 0.3728428/Hartree; Gibbs Free Energy = −11098.813794/Hartree.
